# Haplotype-resolved genomics identifies *cyp19a1a* as a candidate master sex-determining gene in golden trevally (*Gnathanodon speciosus*)

**DOI:** 10.1016/j.isci.2025.113493

**Published:** 2025-09-02

**Authors:** Bin Fan, Jiamin Guo, Caixia Lei, Sen Yang, Zining Meng, Junyao Peng, Yongjian Yang, Yubang Shen, Yuanyou Li, Le Wang

**Affiliations:** 1 Yangjiang Polytechnic, Yangjiang 529500, China; 2 Yangjiang Haina Fisheries Co., Ltd., Yangjiang 529500, China; 3 Key Laboratory of Freshwater Aquatic Genetic Resources, Ministry of Agriculture and Rural Affairs, Shanghai Ocean University, Shanghai 201306, China; 4 Pearl River Fisheries Research Institute, Chinese Academy of Fishery Sciences, Guangzhou 510380, China; 5 College of Food Science and Technology, Guangdong Ocean University Yangjiang Campus, Yangjiang 529500, China; 6 School of Life Sciences, Sun Yat-Sen University, Guangzhou 510275, China; 7 Yangjiang Hongyun Marine Fish Seed Breeding Co., Ltd., Yangjiang 529500, China; 8 College of Marine Sciences, South China Agricultural University, Guangzhou 510642, China; 9 Molecular Population Genetics Group, Temasek Life Sciences Laboratory, Singapore 117604, Singapore

**Keywords:** Ichthyology, Genomics, Developmental genetics, Evolutionary developmental biology

## Abstract

Teleost fish exhibit diverse genetic sex determination systems, offering opportunities to understand its genetic basis and insights into sex chromosome evolution. The golden trevally (*Gnathanodon speciosus*), a commercially important Carangidae species, was studied to uncover its sex determination mechanism. We generated haplotype-resolved, chromosome-level genome assemblies for both sexes and identified a ZW system with a ∼90 kb sex-determining region (SDR) on Chr1. Within the SDR, *cyp19a1a* is uniquely expressed from the W allele prior to gonadal differentiation. A ∼1-kb upstream region of *cyp19a1a* harbors fixed variants between sexes. Reporter gene assays reveal that these variants reduce promoter activity in the Z allele, likely silencing *cyp19a1aZ*. This suggests that allelic diversification led to the emergence of *cyp19a1aW* as the master sex-determining gene. Minimal divergence in the SDR implies a recent origin of sex chromosomes. These findings provide key genomic resources and insights into sex determination evolution in teleosts.

## Highlights

• The haplotypic genome sequences were assembled and annotated
• *cyp19a1a* was identified as the candidate master sex-determining gene
• *cyp19a1a* evolved to be the sex-determining gene through allele diversification
• Minimal sequence divergence was observed between the Z and W chromosomes

## Graphical abstract

**Figure:**
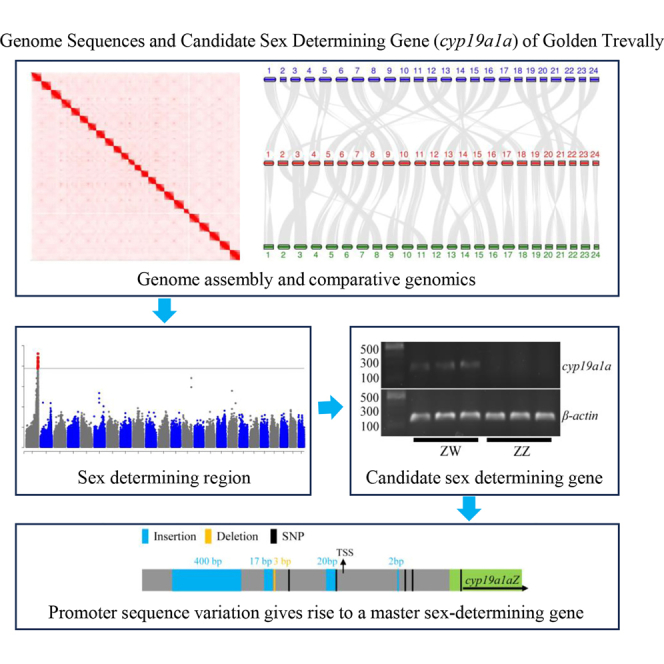

## Introduction

In teleosts, sex determination can be influenced by genetic factors, environmental factors, or their interactions.^1^^,^^2^^,^^3^^,^^4^ In genetic sex determination systems, sex can be governed by either a monogenic or multifactorial basis.^5^ In monogenic systems, numerous genes within the transforming growth factor β (TGF-β) signaling pathway, such as *amh*, *amhr2*, *gsdf*, and *gdf6*, have independently and recurrently evolved as master sex-determining (MSD) genes. This occurs through either gene duplication and neofunctionalization or the formation of new alleles via allelic diversification.^6^^,^^7^^,^^8^^,^^9^^,^^10^ In certain Carangidae fish, the steroidogenic enzyme gene *hsd17b1* has repeatedly evolved as an MSD gene through allelic diversification and is consistently associated with female heterogamety (ZW system).^11^^,^^12^^,^^13^ Notably, the mutations involved either alter the enzyme’s catalytic efficiency due to coding changes or affect its expression pattern and transcript splicing of one allele by modifying regulatory regions.

The emergence of new MSD genes can drive the turnover of sex determination systems and sex chromosomes.^14^^,^^15^ Recent studies in teleosts have shown that, in some cases, the lack of homology between proto-sex chromosomes prevents recombination, triggering the differentiation of sex-linked regions.^7^^,^^10^^,^^15^^,^^16^^,^^17^^,^^18^ However, when MSD genes arise from a single-nucleotide mutation, recombination between sex chromosomes may persist, limiting sequence divergence.^11^^,^^12^^,^^15^ With their frequent shifts in sex determination systems, teleosts offer a unique opportunity to study sex chromosome evolution at various stages of differentiation.

The golden trevally (*Gnathanodon speciosus*) is a marine fish belonging to the family Carangidae.^19^ To date, with the exception of the New Zealand trevally (*Pseudocaranx georgianus*),^20^ all identified sex determination systems in Carangidae fishes have exhibited female heterogamety (ZW system). Furthermore, all discovered MSD genes in this family have been associated with the steroidogenic enzyme gene *hsd17b1*, though the mutations driving its evolution as an MSD gene differ among species.^11^^,^^12^^,^^13^ For example, coding mutations affecting different amino acid residues have been identified in *S. aureovittata*, *S. dumerili*, and *S. quinqueradiata*,^11^^,^^21^ while regulatory mutations around *hsd17b1* have been found in *S. dorsalis*^13^ and *Trachinotus anak*.^12^ A notable exception is the New Zealand trevally, where sex determination follows a male heterogametic (XY) system. In this species, a Y-specific duplication of the steroidogenic enzyme gene *cyp19a1a* has evolved into the MSD gene, highlighting a distinct mechanism from other Carangidae fishes. These findings suggest frequent turnovers of MSD genes within the family. Additionally, in all studied Carangidae species, except for the New Zealand trevally, MSD genes have consistently arisen from single-nucleotide mutations or short sequence variants, resulting in minimal sequence divergence between sex-linked regions.^11^^,^^12^^,^^13^^,^^20^

In this study, we sequenced and assembled high-quality chromosome-level haplotype-resolved genome sequences of the golden trevally, another fish in this family, to investigate the genetic basis of sex determination. We found that *cyp19a1a* evolved into the MSD gene through a different mechanism than in New Zealand trevally. Specifically, a sequence insertion disrupted the promoter of the Z-linked *cyp19a1a* (*cyp19a1aZ*), silencing its expression and driving its evolution into a female-determining gene through allelic diversification. Additionally, we observed minimal sequence divergence in the sex-determining region (SDR) between the sex chromosomes. Our findings provide insights into the emergence of an MSD gene within the ZW sex determination system and highlight the frequent turnover of MSD genes in Carangidae fishes.

## Results and discussion

### Chromosome-level genome assemblies

**Figure 1:**
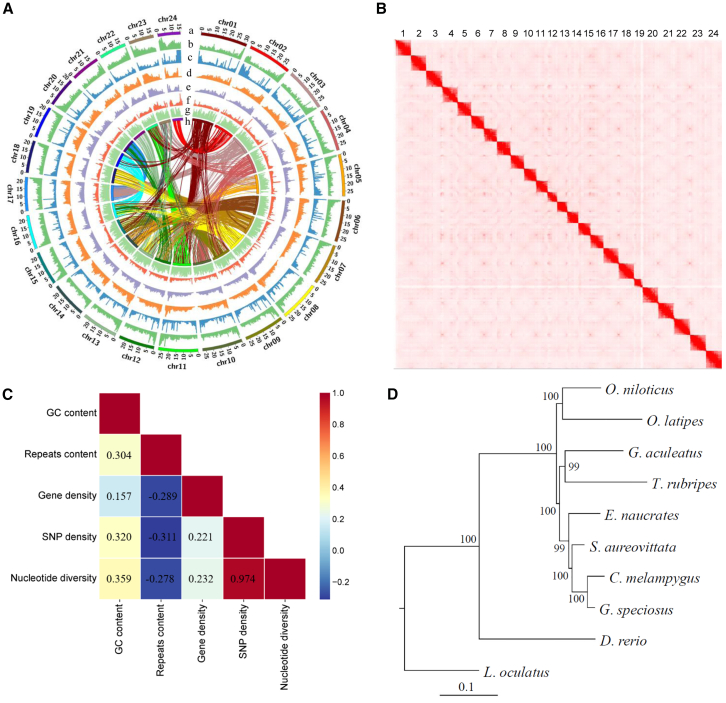
Characterization of the genome sequences and evolution of the golden trevally (A) Distribution of predicted genomic elements throughout the genome in 250-kb nonoverlapping windows (a: pseudochromosomes; b: GC content; c: repeated sequences; d: SNP density; e: nucleotide diversity; f: recombination rates; g: gene density; h: paralogous gene pairs in corresponding chromosomes). (B) Heatmap of the intensity of chromosome interactions in the form of chromosome conformation captured by Hi-C sequencing. Strong individual interaction blocks indicate intrachromosomal interactions. (C) Heatmap of pairwise Pearson’s correlations among genomic elements: GC content, sequence repeat content, gene density, SNP density, and nucleotide diversity. Pearson’s correlation coefficient is shown. (D) Phylogenetic tree showing the genetic relationships among representative teleost species.

The estimated genome size of golden trevally is approximately 562.6 Mb (Figure S1). Using PacBio HiFi sequencing (∼70× coverage), we assembled 246 contigs with a total length of ∼593.0 Mb and an N50 of ∼21.2 Mb for the male sample (Tables S1 and S2). The genome has a GC content of ∼42.7% and an estimated heterogeneity of ∼0.452%. Over 99.7% of the sequences were anchored onto 24 haploid pseudochromosomes using Hi-C chromatin conformation mapping (Figures 1A and 1B; Table S3). The final assembly consists of 64 scaffolds with a scaffold N50 of 27.0 Mb. BUSCO analysis indicated that 98.7% of core genes were complete, with only 1.0% missing (Table S4). Additionally, ∼95.3% of mRNA sequencing reads and ∼98.2% of genome resequencing reads mapped to the genome assembly. To identify the sex-determining gene, we further sequenced and assembled both a hybrid and haplotype-resolved genome sequences from a female sample using ∼60× PacBio HiFi reads and a reference-guided scaffolding strategy. The hybrid female genome exhibited minimal differences (∼595.3 Mb, BUSCO: 98.5%), while the haploid genomes were slightly shorter (∼578.4 and 576.7 Mb) with slightly reduced completeness (BUSCO: 95.0% and 96.0%) compared to the male genome (Tables S2–S4).

### Genome annotation and comparative genomics

We annotated the male genome, identifying ∼99.4 Mb (∼16.8%) as repetitive sequences, with DNA elements and simple repeats comprising 4.65% and 5.28%, respectively (Table S5). GC content was positively correlated with both repeat content and SNP density (Pearson’s *R* > 0.304, *p* < 0.0001), while repeat content was negatively correlated with gene density (Pearson’s *R* = −0.278, *p* < 0.0001) (Figure 1C). Interestingly, repeat content also showed a negative correlation with SNP density (Pearson’s *R* = −0.311, *p* < 0.0001), which is likely due to low mapping quality in highly repetitive regions for short-read sequencing, resulting in reduced coverage and missed SNPs.^22^ A total of 23,280 protein-coding genes were predicted, with 82.7% homologous to known proteins or domains in the protein family database Pfam v.36.0.^23^

**Figure 2:**
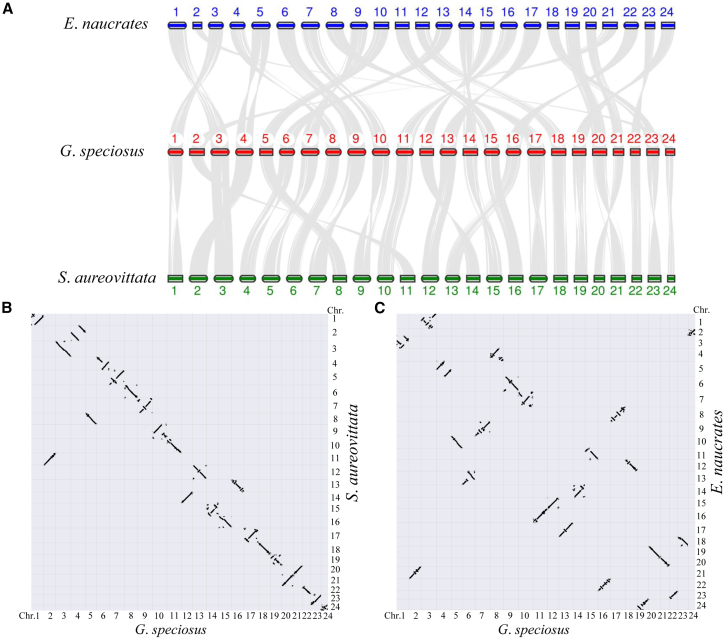
Conserved genomic synteny between golden trevally and carangiform fishes (A) Genomic synteny between golden trevally (*G. speciosus*) and the live sharksucker (*E. naucrates*) and between golden trevally and the Japanese yellowtail jack (*S. aureovittata*). Chromosomes are specified by numeric values. (B) Genomic synteny between golden trevally and the Japanese yellowtail jack, revealed by Oxford Grid dot plots. (C) Genomic synteny between golden trevally and the live sharksucker, revealed by Oxford Grid dot plots.

Phylogenetic analysis placed the golden trevally within a distinct Carangidae clade, showing its closest relationship to the bluefin trevally (*Caranx melampygus*) from a different genus (Figure 1D). We observed a high level of genomic synteny between the golden trevally and the live sharksucker (*Echeneis naucrates*), as well as between the golden trevally and the Japanese yellowtail jack (*S. aureovittata*) (Figure 2A). However, intrachromosomal rearrangements were observed throughout all chromosomes (Figures 2B and 2C). No chromosome fusion or fission events were detected between the studied species (Figures 2B and 2C). Genomic synteny of paralogous genes resulting from the most recent whole-genome duplication event was clearly detected between chromosomes in golden trevally, including the pairs Chr16 vs. Chr19, Chr2 vs. Chr24, and Chr11 vs. Chr22 (Figure 1A).

### Mapping the SDR

**Figure 3:**
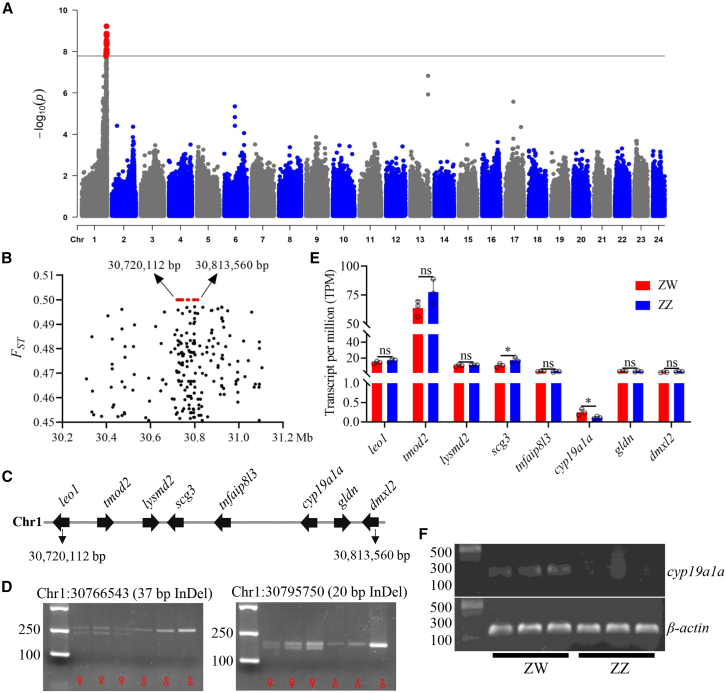
*cyp19a1a* is the candidate master sex-determining gene in golden trevally (A) A single sex-determining locus located at Chr1 is identified in the genome-wide association study. The significance cutoff value, in terms of −log10 (*p* value) of 7.789, is indicated with a solid horizontal line. (B) Distribution of *F*_*ST*_ values of SNPs around the sex-determining locus, where SNPs with fixed differences between females and males are indicated with red solid dots and genomic positions. (C) Eight protein-coding genes are predicted within the sex-determining region (SDR). Gene orientations and names are indicated in the diagram. (D) Two indels within the SDR, which are fixed between females and males in the entire mapping population, are developed as sex markers. (E) Expression of genes within the SDR in trunks at 30 days post-fertilization (dpf) between ZW and ZZ genotypes revealed by transcriptome sequencing (*n* = 3 per genotype, and data are represented as mean ± SEM, with ∗ indicating *p* < 0.05 for two-tailed Student’s t test). (F) Expression of *cyp19a1a* in the samples, as shown in (E), detected by nested reverse-transcription PCR (nested RT-PCR), with β-actin as the reference gene.

With an average of ∼21.5× coverage of sequencing reads per individual (Figure S2), a total of 4,258,752 genome-wide biallelic variants were genotyped across a sex-balanced wild population comprising 30 females and 30 males (see STAR Methods). Whole-genome-wide association study identified a single locus on Chr1 that showed a significant association with phenotypic sex (Figures 3A, S3, and S4). We estimated *F*_*ST*_ values for individual variants around the locus, which showed that 22 SNPs and eight short InDels within a ∼90-kb region (Chr1: 30,720,112-30,813,560) were fixed between the sexes, while genetic variants in the flanking regions were not completely linked with sex (Figure 3B). The sex-specific variants were heterozygous in all females and homozygous in all males, indicating a female heterogametic or ZW sex-determining system. Notably, SNP density was lower in the flanking regions than the SDR (Figure 3B). It is likely caused by suppressed recombination in the SDR, which leads to accumulation of mutations.^7^^,^^18^^,^^24^ Eight protein-coding genes were predicted within the SDR of the male genome (Figure 3C), of which only *cyp19a1a* is functionally linked to TGF-β signaling, steroidogenesis, or sex determination in vertebrates. Five sex-specific SNPs were in coding sequences, with one nonsynonymous SNP located in the first exon of *cyp19a1a* (Table S6). Notably, eight sex-specific variants were clustered within a ∼1-kb region located ∼300 bp upstream of *cyp19a1a* coding sequences (Table S6), indicating ongoing sequence divergence or gradual degeneration in this region. Sequence analysis of the SDR in the haploid ZW genomes confirmed that the only difference in coding sequences of the eight genes between the Z and W alleles was the nonsynonymous SNP in *cyp19a1a*, with no evidence of gene gain or loss.

### *cyp19a1a* shows female-biased expression before gonadal differentiation

To identify the MSD gene, we examined gene expression between ZZ and ZW genotypes before gonadal differentiation. We designed two genetic markers within the SDR to identify genetic sex, which were fixed between sexes in the mapping population (Figure 3D). In the studied Carangidae fishes, gonadal differentiation between sexes occurs around 40 days post-fertilization (dpf),^11^^,^^25^ indicating that the critical stage for sex determination occurs before this time. Similarly, in golden trevally, we observed that gonads in both ZW and ZZ genotypes remained undifferentiated at 30 dpf (Figure S5A). Transcriptomic analysis of trunk tissues containing gonads at 30 dpf showed that only *cyp19a1a* exhibited significant female-biased expression (fold change ∼2.2), despite a low basal expression with a transcripts per million value of ∼0.5. In contrast, only one gene, *scg3* (*secretogranin III*), displayed male-biased expression (fold change ∼1.6) (Figures 3E and S5B). Nested reverse-transcription PCR and qPCR confirmed that *cyp19a1a* transcripts were expressed in ZW but not ZZ samples (Figures 3F and S5C). Although we cannot rule out the possibility that *scg3* may be a candidate male-biased MSD gene in a ZW sex determination system, similar to the role of *dmrt1* in birds,^26^ it is less likely than *cyp19a1a* to function as an MSD gene. This is because previous studies suggest that *scg3* is more likely involved in sexual behavior and reproduction rather than primary sex determination in vertebrates.^27^^,^^28^^,^^29^

Further analyses on ovary and testis transcriptomes and expression revealed two *cyp19a1a* transcripts. In ZW ovary, one transcript from the *cyp19a1a* locus containing an intact open reading frame encodes a functional protein that is conserved among vertebrates (we infer that this is from the *cyp19a1aW* allele) (Figures S6 and S7). The other transcript in the ZW ovary displayed partial overlap with the *cyp19a1aW* transcript, contained premature termination codons, and demonstrated no homology to the W-linked transcript in terms of the predicted coding sequences (this is probably from the *cyp19a1aZ* allele) (Figure S8), and, indeed, in ZZ testis, only this *cyp19a1a* transcript was identified. We aligned both transcripts to the locus and found that the two transcripts were transcribed from opposite strands and only partially complementary, lacking homology in predicted translation products (Figures S9A and S9B). These findings were further confirmed by qPCR (Figure S9C).

The female-biased expression of *cyp19a1a* in golden trevally is consistent with the that of typical MSD genes in the ZW sex determination system, where the expression of Z-linked transcripts or the function of gene products from the Z allele is compromised.^11^^,^^12^^,^^30^ In short, sex determination is governed by the presence of the dominant W-linked gene, *cyp19a1aW*, in golden trevally. In both zebrafish and female Nile tilapia with an XX genotype, disruption of *cyp19a1a* has been shown to lead to male development and female-to-male sex reversal, respectively.^31^^,^^32^ All these data strongly support the hypothesis that *cyp19a1a* is the most likely candidate MSD gene in golden trevally.

### *cyp19a1a* evolves into an MSD gene through allelic diversification

**Figure 4:**
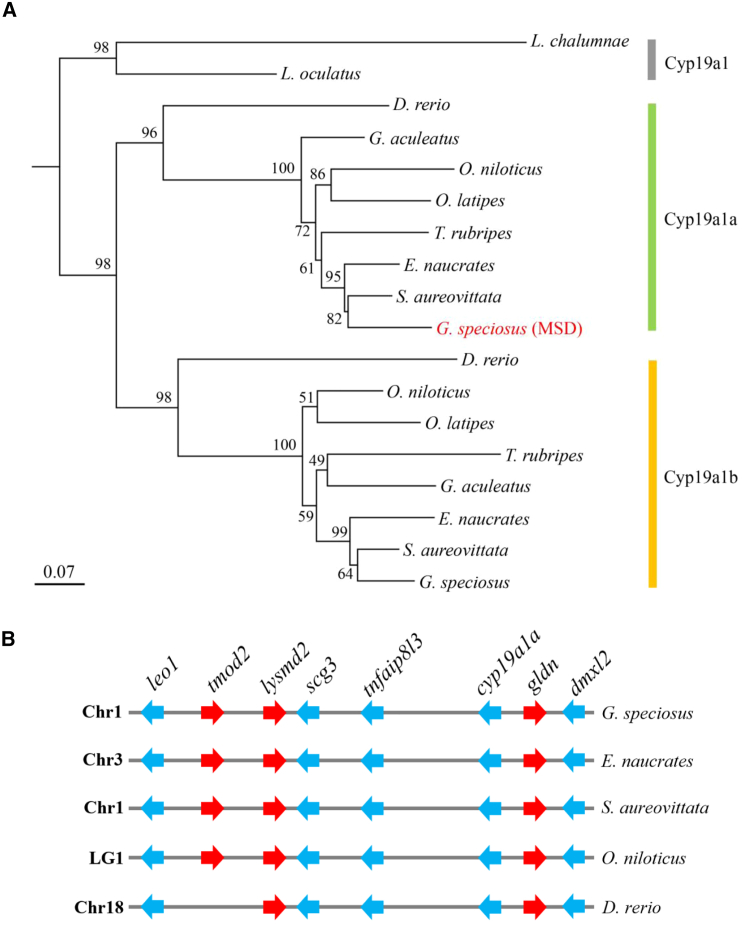
The sex-determining locus is conserved across teleost species (A) A phylogenetic tree constructed using Cyp19a1 protein sequences from teleost species under the JTT+G4 amino acid substitution model. Bootstrap values from 1,000 replicates are shown near the branches. (B) Genes and their genomic organization within the SDR are conserved across teleost species.

To investigate the evolutionary origin of the candidate MSD gene, we analyzed both female and male genome sequences, including their corresponding haploid assemblies, to identify homologs. Our search revealed a single copy of *cyp19a1a* and *cyp19a1b* in both genomes (Figure 4A). Comparative genomic analysis further showed that genes within the SDR were highly conserved across teleost species (Figure 4B). These data suggest that *cyp19a1a* evolved into the MSD gene in golden trevally through allelic diversification affecting gene expression rather than through gene duplication. The emergence of the MSD gene in golden trevally follows the evolutionary patterns observed in other Carangidae fishes,^11^^,^^12^^,^^13^^,^^21^ with the exception of New Zealand trevally, where the MSD gene likely arose from gene duplication followed by translocation.^20^

Given the diverse sex determination mechanisms in Carangidae fishes,^11^^,^^12^^,^^13^^,^^20^ our findings further emphasize the frequent turnover of sex chromosomes and MSD genes in this family. Notably, in New Zealand trevally, a Y-specific duplication of the same gene evolved into a male-determining factor, though the precise mechanism remains unclear.^20^ In vertebrates, transitions between XY and ZW sex determination systems have been documented across various species, particularly in fish and amphibians.^33^^,^^34^^,^^35^ These shifts often result from changes in sex-determining genes and the underlying genetic mechanisms regulating sex determination. These findings suggest that transitions between XY and ZW systems may occur more readily than previously thought, potentially driven by the emergence of a dominant allele in a key sex-determining gene.

Aromatase deficiency in humans, caused by heterozygous *cyp19a1a* mutations, can lead to various disorders.^36^^,^^37^^,^^38^ This raises an important question: how is aromatase function maintained in male golden trevally, given that the sole copy of *cyp19a1a* is silenced? To investigate this, we analyzed *cyp19a1b* expression in transcriptomes and detected basal expression in the testis (Figure S10A). Additionally, *cyp19a1b* expression in the trunks of ZZ genotypes was comparable to *cyp19a1a* expression in ZW genotypes at 30 dpf (Figure S10B). These findings suggest that the loss of *cyp19a1a* function may be compensated by its paralog *cyp19a1b*, despite their significant functional divergence (Figure 4B).

### A promoter insertion suppresses *cyp19a1aZ* expression

**Figure 5:**
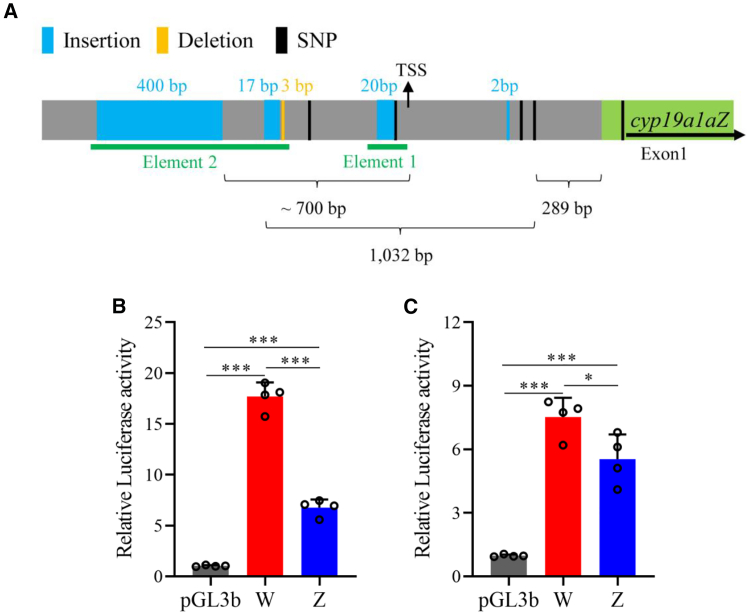
Genetic variants in the promoter region of cyp19a1aZ are associated with its compromised expression (A) Schematic representation of genetic variants, including SNPs, insertions, and deletions, which are fixed between females and males in the entire mapping family. The distances between variant pairs, the positions of predicted transcription start site (TSS), and two genomic elements with large sequence variations are indicated. (B) Element 1, shown in (A), which contains a 20-bp insertion and a downstream SNP in the Z allele, significantly reduces promoter activity in luciferase assays compared to the W allele. (C) Element 2, shown in (A), which contains a 400-bp insertion, a 17-bp insertion, and a 3-bp deletion in the Z allele, leads to a slight reduction in promoter activity compared to the W allele in luciferase assays. *n* = 4 for each treatment, and data are represented as mean ± SEM, with ∗ indicating *p* < 0.05 and ∗∗∗ indicating *p* < 0.001 (two-tailed Student’s t test).

To identify the genetic variant responsible for the silencing of *cyp19a1aZ*, we examined the surrounding sequences. Apart from several simple sequence repeats distributed across the SDR, no major structural variants were detected between the Z and W alleles, except for a ∼400 bp insertion located ∼700 bp upstream of the predicted transcription start site (TSS) of cyp19a1aZ (Figure 5A). This insertion is sex specific across the mapping population (Figure S11A) and consists of a highly repetitive tandem repeat motif of 26 nucleotides (Figure S11B). Additionally, this structural variant locates ∼230 bp upstream of a ∼1-kb region containing eight sex-specific variants mentioned earlier (Figure 5A). Within this region, we identified several sequence variants exclusive to the Z allele, including 17-bp, 20-bp, and 2-bp insertions, as well as a 3-bp deletion. Notably, the 20-bp insertion, previously used as sex marker, and its adjacent sex-specific SNP are located in close proximity to the predicted core promoter of *cyp19a1a* (Figure 5A). We hypothesized that genetic variants in the promoter region of *cyp19a1aZ* contribute to its silencing. Since single SNPs are generally less impactful on gene expression than larger structural variants, we focused on the latter. To test their effects, we separately cloned the W and Z alleles of two genomic regions into a luciferase reporter system. The first region (element 1) contained the 20-bp insertion and its adjacent SNP, while the second region (element 2) included the 400-bp and 17-bp insertions along with the 3-bp deletion (Figure 5A). Luciferase assays revealed that, for element 1, the Z allele significantly decreased expression compared to the W allele, though both alleles enhanced reporter gene expression overall (Figure 5B). A similar trend was observed for element 2, but the reduction in expression caused by the Z allele was less pronounced (Figure 5C). Although we cannot rule out the possibility that other sex-specific variants may also contribute to the silencing of *cyp19a1aZ*, they are unlikely to be the primary causal mutations, as they are not located within the predicted regulatory region of the gene. Notably, the 37-bp insertion in the Z allele, used as a sex marker, is located approximately 1.4 kb upstream of the *cyp19a1aZ* TSS, potentially acting as a promoter element for this nonfunctional transcript.

Genetic variants within element 2 likely have a relatively minor effect and are unlikely to be the primary causal mutations. In contrast, variants located near the predicted core promoter sequences are more likely responsible for the silencing of *cyp19a1aZ*. Among them, the 20-bp insertion is the strongest candidate as the primary causal mutation or a trigger for the turnover and emergence of the MSD gene. Therefore, the emergence of the MSD gene in golden trevally is most likely the result of a single mutation, similar to the allelic diversification observed in many teleost species where MSD genes have evolved.^7^^,^^11^^,^^12^^,^^13^^,^^39^ However, it is also possible that the silencing of *cyp19a1aZ* results from a mutated haplotype containing multiple sex-specific variants, similar to the observation in medaka species *Oryzias luzonensis*.^10^ Overall, our findings suggest that sequence variations have disrupted the promoter function of *cyp19a1aZ*, leading to its silencing and ultimately facilitating the emergence of the MSD gene through allelic diversification.

### Limited sequence divergence despite recombination suppression in the SDR

We separately mapped sequencing reads from ZW and ZZ genotypes to the sex chromosomes and found little difference in sequence depth across the sex chromosomes. Additionally, repeat sequence annotation revealed little variation in repeat content between the W and Z chromosomes. Within and around the SDR, both sequence divergence and repeat content between Z and W alleles remained minimal (Figures S12 and S13), indicating limited differentiation between the sex chromosomes. As noted earlier, the most substantial evidence of genetic variant accumulation or gradual sequence degeneration appears to be limited to the putative promoter region, spanning just over 1 kb and containing nine sex-specific SNPs and InDels. However, recombination suppression extended across the SDR and its adjacent flanking regions, covering approximately 300 kb, significantly beyond the SDR itself (Figure S14).

Despite limited differentiation, the pattern of sex chromosome differentiation in golden trevally differs from that observed in *Seriola* fishes and pufferfish species, where MSD genes originate from a single SNP, and the sex chromosomes have remained undifferentiated for at least five million years due to frequent recombination around the MSD gene.^11^^,^^15^ In golden trevally, the higher degree of differentiation, compared to species with undifferentiated sex chromosomes aside from a single sex-determining SNP, further supports our conclusion that the emergence of the MSD gene in this species is driven by a structural sequence variant.

Sex chromosome differentiation is more common in species where MSD genes arise from large structural variations rather than single point mutations and occur over an extended timescale.^14^^,^^18^^,^^40^ In such species, the emergence of MSD genes disrupts sequence homology, leading to recombination suppression between the emerging Z and W (or X and Y) chromosomes, which in turn facilitates the accumulation of mutations. These two processes interact dynamically, progressively driving sex chromosome divergence and distinguishing them from their homologous counterparts over time.^17^^,^^24^^,^^41^ In golden trevally, the limited sequence divergence despite recombination suppression suggests that the MSD gene arose relatively recently and that the sex chromosomes are still in the early stages of differentiation, similar to the pattern observed in fighting fish.^7^

In conclusion, our study provides a comprehensive genomic analysis of the golden trevally, revealing a ZW sex determination system driven by a recently evolved MSD gene derived from the vertebrate female sex determination pathway. Female sex determination in this species is associated with the silencing of the *cyp19a1a* gene, a member of the steroidogenic enzyme family. Specifically, one allele, *cyp19a1aZ*, is silenced through sequence variants in its promoter region, thereby promoting its role in female sex determination. Despite evidence of recombination suppression and accumulation of fixed mutations, the minimal sequence divergence between the W and Z alleles within the SDR indicates that the sex chromosomes are still in the early stages of differentiation. Notably, although the MSD genes in New Zealand trevally and golden trevally are paralogs, their functional roles may differ. Comparing these two systems offers a valuable opportunity to explore how homologous genes can lead to distinct sex determination mechanisms, thereby enhancing our understanding of the plasticity of conserved top-tier sex determination signaling cascades.

### Limitations of the study

Functional verification of the candidate sex-determining gene *cyp19a1a* through gene knockout and transgenesis has not been performed due to the absence of established platforms, which remain a significant challenge for marine fish species. The phased female haplotype genome assemblies were constructed solely from fragmented HiFiasm assemblies using a reference-guided approach, which may introduce phase-switch errors across the genome. Future studies incorporating Hi-C or trio-binning data will help improve the accuracy and completeness of haplotype-resolved female genome assemblies.

## Resource availability

### Lead contact

Further information and requests should be directed to the lead contact Dr. Le Wang (lewang.wang@hotmail.com).

### Materials availability

This study did not generate new unique reagents.

### Data and code availability

• Data: Data reported in this paper will be shared by the lead corresponding author upon request. Raw sequencing reads for transcriptome sequencing and whole-genome resequencing are archived in China National GeneBank (CNGB, https://db.cngb.org/cnsa/) Database: BioProject CNP0005127. Raw sequencing reads for genome assembly and the chromosome-level genome sequence are archived in the National Genomics Data Center (NGDC, https://ngdc.cncb.ac.cn/) Database: BioProject PRJCA022499 and in the Genome Warehouse of NGDC Database: GWHEQWS00000000.
• Code: No new code has been generated in this work.
• Additional information: Any additional information required to reanalyze the data reported in this paper is available from the lead corresponding author upon request.

## Acknowledgments

We thank the anonymous fishermen for the collection of samples. This work was supported by 10.13039/501100021171Guangdong Basic and Applied Basic Research Foundation (grant number: 2021A1515011123) and Guangdong Modern Agricultural Industrial Park (grant number: GDSCYY2020-011) of China.

## Author contributions

B.F.: methodology, data curation, formal analysis, and funding acquisition. J.G.: methodology, visualization, and formal analysis. C.L.: methodology and formal analysis. S.Y.: methodology and visualization. Z.M.: methodology. J.P.: sample preparation. Y.Y.: sample preparation. Y.S.: methodology. Y.L.: supervision and funding acquisition. L.W.: conceptualization, supervision, writing – original draft, and writing – review and editing.

## Declaration of interests

The authors declare no competing interests.

## STAR★Methods

### Key resources table

**Table:** 

REAGENT or RESOURCE	SOURCE	IDENTIFIER
**Chemicals, peptides, and recombinant proteins**		

DNase I	Roche	Cat#04716728001
Super M-MuLV Reverse Transcriptase Kit	Sangon Biotech	Cat#B110022-0002
pGL3-Basic plasmid	Promega	Cat#E1751
pRL Renilla plasmid	Promega	Cat#E2231
Lipofectamine 3000	Thermo Fisher	Cat#L3000015
TRIzol™ Reagent	Thermo Fisher	Cat#15596018
PCR master mix	Sangon Biotech	Cat#B639295
Truseq DNA PCR free Library Preparation Kit	Illumina	Cat#20015963
TruSeq Stranded mRNA Library Prep Kit	Illumina	Cat#RS1222001
SYBR® Green Real-time PCR Master Mix	TOYOBO	Cat#QPK-101
Dual-Luciferase® Reporter Assay System	Promega	Cat#E1910

**Deposited data**		

PacBio sequencing data	This study	NGDC: PRJCA022499
Illumina sequencing data	This study	CNGBdb: CNP0005127
Hi-C sequencing data	This study	CNGBdb: CNP0005127
Transcriptome sequencing data	This study	NGDC: PRJCA022499
Resequencing data	This study	CNGBdb: CNP0005127
Genome assembly data	This study	NGDC: GWHEQWS00000000
Genome assembly data	This study	NGDC: GWHFPUH00000000.1
*Echeneis naucrates* data	Venkatesh et al. (unpublished)	NCBI: GCA_900963305.2
*Seriola dorsalis* data	Purcell et al.^13^	NCBI: GCA_002814215.1
*Seriola aureovittata* data	Li et al.^21^	NCBI: GCF_021018895.1
*Caranx melampygus* data	Pickett et al.^42^	NCBI: GCA_019059645.1
*Trachinotus anak* data	Zhang et al.^43^	NCBI: GCA_022709315.1
*Lepisosteus oculatus* data	Braasch et al.^44^	NCBI: GCA_000242695.1
*Danio rerio* data	Howe et al.^45^	NCBI: GCF_000002035.6
*Oreochromis niloticus* data	Conte et al.^46^	NCBI: GCF_001858045.2
*Oryzias latipes* data	Kasahara et al.^47^	NCBI: GCF_002234675.1
*Gasterosteus aculeatus* data	Jones et al.^48^	NCBI: GCA_000180675.1
*Takifugu rubripes* data	Aparicio et al.^49^	NCBI: GCA_000180615.2

**Software and algorithms**		

GenomeScope (v2.0)	Ranallo-Benavidez et al.^50^	https://github.com/tbenavi1/genomescope2.0/
Hifiasm (v0.16.0)	Cheng et al.^51^	https://github.com/chhylp123/hifiasm/
Pilon (v1.19)	Walker et al.^52^	https://github.com/broadinstitute/pilon/
Stacks (v2.45)	Rochette et al.^53^	https://catchenlab.life.illinois.edu/stacks/
Juicer (v1.5)	Durand et al.^54^	https://github.com/aidenlab/juicer/
3D-DNA (v180922)	Dudchenko et al.^55^	https://github.com/aidenlab/3d-dna/
Juicebox (v2.3.0)	Durand et al.^56^	https://github.com/aidenlab/Juicebox/
RaGOO (v1.1)	Alonge et al.^57^	https://github.com/malonge/RaGOO/
BUSCO (v5.2.2)	Simão et al.^58^	https://busco.ezlab.org/
RepeatMasker (v3.3.0)	Chen^59^	https://github.com/rmhubley/RepeatMasker/
Repbase (v20170127)	Jurka et al.^60^	https://www.girinst.org/repbase/
RepeatModeler (v2.0.3)	Flynn et al.^61^	http://www.repeatmasker.org/
Tandem Repeat Finder (v4.09)	Benson^62^	https://tandem.bu.edu/trf/trf.html/
Braker (v3.0.3)	Brůna et al.^63^	https://github.com/Gaius-Augustus/BRAKER/
Maker (v2.31.9)	Holt & Yandell^64^	https://github.com/Yandell-Lab/maker/
SNAP (v2013)	Korf^65^	https://github.com/KorfLab/SNAP/
GeneMark (v1.0)	Brůna et al.^66^	https://exon.gatech.edu/
Augustus (v3.2.3)	Stanke et al.^67^	https://bioinf.uni-greifswald.de/augustus/
Ortholog-finder (v2.4.0)	Horiike et al.^68^	https://github.com/davidemms/OrthoFinder/
MUSCLE (v3.8.1)	Edgar^69^	https://www.drive5.com/muscle/
trimAl (v1.2)	Capella-Gutiérrez et al.^70^	https://github.com/inab/trimal/
IQ-TREE (v1.5.5)	Minh et al.^71^	http://www.iqtree.org/
ModelFinder (v1.5.5)	Kalyaanamoorthy et al.^72^	http://www.iqtree.org/
MCscan (v0.8.12)	Tang et al.^73^	https://github.com/tanghaibao/mcscan/
LDhat (v2.2a)	Auton & McVean^74^	https://github.com/auton1/Ldhat/
BWA-mem (v0.7.17)	Li & Durbin^75^	https://bio-bwa.sourceforge.net/
GATK (v4.2.2.0)	McKenna et al.^76^	https://gatk.broadinstitute.org/hc/en-us/
GAPIT (v3.0)	Wang & Zhang^77^	https://github.com/jiabowang/GAPIT/
VCFtools (v0.1.15)	Danecek et al.^78^	https://vcftools.sourceforge.net/
Trinity (v2.1.1)	Grabherr et al.^79^	https://github.com/trinityrnaseq/trinityrnaseq/
BBmap (v37.09)	Bushnell et al.^80^	https://github.com/BioInfoTools/BBMap/
STAR (v2.5.2b)	Dobin et al.^81^	https://github.com/alexdobin/STAR/
HTSeq-count (v0.9.1)	Anders et al.^82^	https://github.com/simon-anders/htseq/
Pfam (v36.0)	Mistry et al.^23^	http://pfam.xfam.org/

### Experimental model and subject details

#### Ethical approval

All fish handling procedures followed the guidelines of the Animal Ethics Committee of Yangjiang Polytechnic, Guangdong, China (Approval No. 2021DW004).

#### Animals

Fish used for genome sequencing and transcriptome sequencing were collected from the fish farm of Yangjiang Hongyun Marine Fish Seed Breeding Co., Ltd., Yangjiang 529500, China. Fish used for association analysis were randomly collected from South China Sea, China. The fish used in this study ranged in age from 30 days post-fertilization to two years, with individuals over approximately one year old being sexed based on phenotype. Fish collected from hatcheries were reared under standard aquaculture conditions. All animals were euthanized by decapitation.

### Method details

#### Genome sequencing and assembly

A mature male golden trevally at two years old were chosen for genome sequencing using PacBio HiFi and Hi-C (chromosome conformation capture) sequencing technologies, while a female was only selected for PacBio sequencing. Genomic DNA isolated from muscle tissue of each sample was used to construct a 15-kb insert PacBio library. Approximately 40 Gb and 35 Gb of raw reads were obtained for male and female samples, respectively (Table S1). A 350-bp short insert library was also constructed for Illumina sequencing and ∼58 Gb of 2 × 150 bp raw reads were obtained for male sample. Illumina reads were cleaned using BBmap.^80^ Genome size and heterozygosity were estimated with GenomeScope v2.0^50^ with a k-mer value of 17. Heterozygosity was calculated as the fraction of base positions that differ between the two haplotypes, inferred from the proportion of heterozygous k-mers. A Hi-C library was prepared following a previous method,^83^ with some modifications according to our previous study.^84^ The library was sequenced for 2 × 150 bp reads using the NovaSeq 6000 platform (Illumina, USA) and ∼120 Gb reads were generated. The PacBio HiFi reads were cleaned and assembled using the Hifiasm v0.16.0 assembler^51^ for both hybrid and haploid genome sequences with default parameters. Assembled contigs were polished using Pilon v1.19^52^ with Illumina reads. Hi-C reads were initially cleaned with the program *process_shortreads* in Stacks v2.45 package.^53^ Subsequently, the cleaned reads were used to anchor the assembled contigs into scaffolds sequentially using Juicer v1.5^54^ and 3D-DNA v180922 pipelines.^55^ The scaffolds were then manually curated using Juicebox v2.3.0,^56^ with a prior setting of 24 haploid chromosomes.^85^ Chromosome-level genome sequences for the female sample were assembled using RaGOO v1.1^57^ through reference-guided scaffolding, with the male genome sequence serving as the reference. BUSCO v5.2.2^58^ was utilized to evaluate the completeness of the genome sequences based on the database actinopterygii_odb10.

#### Genome annotation

The program RepeatMasker v3.3.0^59^ was utilized to annotate repetitive sequences, based on both the Repbase database v20170127^60^ and a custom repeat library constructed using RepeatModeler v2.03.^61^ Short tandem repeats were annotated using Tandem Repeat Finder v4.09.^62^ These repetitive sequences were merged to create a nonredundant annotations of repetitive sequences in the genome. Protein-coding genes were predicted based on the protein homology of related species. In brief, protein sequences from *Echeneis naucrates* (NCBI: GCA_900963305.2), *S. dorsalis*,^13^ *S. aureovittata*,^21^ *Caranx melampygus*^42^ and *T. anak*^43^ were obtained from the National Center for Biotechnology Information database (NCBI) for prediction using the program Braker v3.0.3.^63^ The nonredundant transcript dataset assembled below was then used as supporting evidence for annotations. The program Maker v2.31.9 pipeline^64^ was utilized to integrate the predictions from Braker and the evidence from transcript data. Predicted gene models were iteratively trained using SNAP v2013,^65^ GeneMark v1.0,^66^ and Augustus v3.2.3^67^ for three iterations. Predicted gene models that contained transposable element (TE) domains and lacked support from transcripts were subsequently filtered out and removed.

#### Evolutionary analysis

For phylogenetic analysis, protein sequences of the following species were extracted from NCBI: spotted gar (*Lepisosteus oculatus*),^44^ zebrafish (*Danio rerio*),^45^ Nile tilapia (*Oreochromis niloticus*),^46^ medaka (*Oryzias latipes*),^47^ stickleback (*Gasterosteus aculeatus*),^48^ fugu (*Takifugu rubripes*),^49^ live sharksucker (*E. naucrates*) (NCBI: GCA_900963305.2), the Japanese yellowtail jack (*S. aureovittata*),^21^ and bluefin trevally (*C. melampygus*).^42^ One-to-one orthologues across these species were identified through pairwise blast searches using Ortholog-finder v2.4.0.^68^ Subsequently, individual orthologs were aligned using MUSCLE v3.8.1^69^ and then refined using trimAl v1.2.^70^ A total of 3,358 aligned orthologous protein sequences were concatenated to construct a phylogenetic tree using IQ-TREE v1.5.5,^71^ employing the JTT+F+R5 substitution model determined by ModelFinder^72^ implemented in IQ-TREE v1.5.5.^71^ Homologous chromosome blocks between and within species of interest were detected using the MCscan v0.8.12 pipeline,^73^ utilizing whole genome-wide protein-coding genes as input. Distribution patterns of recombination rate along individual chromosomes, quantified as ρ = 4Ner per kilobase, were estimated using LDhat v2.2a.^74^

#### Mapping the sex determining gene

To map the SD locus, wild samples consisting of 30 females and 30 males were randomly collected from the South China Sea. Genomic DNA was extracted from the fin tissue using TIANamp Marine Animals DNA Kit (Tiangen, China). DNA libraries with an insert size of 500 bp were constructed using Truseq DNA PCR free Library Preparation Kit (Illumina, USA), followed by sequencing to generate 2 × 150 bp reads using NovaSeq 6000 platform (Illumina, USA). Raw reads were filtered with *process_shortreads* in Stacks v2.45.^53^ Cleaned reads were then aligned to the reference genome using BWA-mem v0.7.17,^75^ with default parameters. Variants were discovered and called using the best practices workflow of Picard/GATK using GATK toolkit v4.2.2.0.^76^ SNPs and indels were filtered with the following parameters: ‘QD less than 2.0 || FS greater than 60.0 || MQ less than 40.0 || MQRankSum <−12.5 || ReadPosRankSum <−8.0 || SOR greater than 4.0’ and ‘QD less than 2.0 || FS greater than 200.0 || ReadPosRankSum <−20.0 || SOR greater than 10.0’, respectively. A total of 15,513,443 variants were discovered and genotyped. We further filtered the dataset by removing variants with a minor allele frequency of <0.05 and missing genotypes of >0.05. Association between genotypes and phenotypic sex was assessed using the R package GAPIT v3.0^77^ under a mixed linear model (MLM). Additionally, an F_ST_ scan was conducted between females and males using VCFtools v0.1.15.^78^

#### Transcriptome sequencing and analysis

Total RNA was extracted from liver, eye, and testis of the same fish used for genome sequencing and assembly using TRIzol Reagent (Thermo Fisher Scientific, USA). In addition, total RNA was also extracted from the ovary of a female fish of the same age as the male individual. Subsequently, mRNA libraries were prepared using the TruSeq Stranded mRNA Library Prep Kit (Illumina, USA). The mRNA libraries were sequenced to generate 2 × 150 bp reads using the NovaSeq 6000 platform (Illumina, USA). Transcriptome sequencing reads were cleaned using the program *process_shortreads* in Stacks v2.45^53^ with default parameters. Transcripts were first assembled for each individual samples using Trinity v2.1.1.^79^ Subsequently, transcripts from different samples were merged and redundancy was then reduced using BBmap v37.09^80^ to generate a nonredundant transcript dataset used for predicting gene models.

Fish samples at early developmental stages were collected for histological analysis to determine the gonadal differentiation status by histology according to our previous study.^12^ DNA was extracted from heads and then used for genotyping using sex markers, while trunks were used for RNA isolation and transcriptome sequencing (*n* = 3 for each genotype). To identify differentially expressed genes (DEGs), transcriptome sequencing reads were aligned to the reference genome using STAR v2.5.2b^81^ with default parameters. Counts of uniquely aligned reads were estimated using HTSeq-count v0.9.1^82^ against the annotations of predicted gene models. The relative expression of genes was further measured using transcripts per million (TPM).

#### Examining gene expression pattern

The expression of *cyp19a1a* in trunk samples was analyzed using nested reverse transcription PCR (Nested RT-PCR) and quantified by quantitative real-time PCR (qPCR), following our previous study.^86^ In brief, 2 μg of total RNA from each sample was treated with DNase I (Roche, Switzerland) to remove genomic DNA contamination. Subsequently, the RNA was reverse transcribed into cDNA using the Super M-MuLV Reverse Transcriptase Kit (Sangon Biotech, China). Gene- or transcript-specific primers were designed using Primer3.^87^ For both assays, two rounds of PCR were performed. Briefly, the first-round PCR was conducted using outer primers for 16 cycles in a 25 μL reaction containing 2× PCR master mix (Sangon Biotech, China). The second-round PCR was performed with inner primers, using 2 μL of the first-round PCR product as the template, with 30 cycles for Nested RT-PCR and 35 cycles for qPCR. For Nested RT-PCR, the second-round PCR conditions were identical to those of the first round. The qPCR reactions were conducted using SYBR Green Real-time PCR Master Mix (TOYOBO, Japan) on an ABI 7900HT Fast Real-Time PCR System (ABI, USA). The housekeeping gene *β-actin* was used as a reference for Nested RT-PCR and to normalize gene expression in qPCR using the 2^−ΔΔCT^ method, following our previous approach.^86^ Three biological replicates, each with three technical replicates, were utilized for qPCR analysis.

#### Reporter gene assay

Different alleles of the genomic elements of interest were amplified and cloned into the multiple cloning sites of the pGL3-Basic (pGL3b) plasmid (Promega, USA), which contains a basic SV40 promoter. Reporter gene expression was analyzed in an Asian seabass epithelial-like cell line^88^ cultured in a 24-well plate with Leibovitz’s L-15 medium. Each constructed plasmid (300 ng) was co-transfected with the pRL Renilla vector (200 ng) (Promega, USA) using Lipofectamine 3000 (Thermo Fisher Scientific, USA) when cells reached ∼80% confluency. At 48 h post-transfection, luciferase activities were measured using the Dual-Luciferase Reporter Assay System (Promega, USA) on a Tecan Spark Plate Reader (Tecan, Switzerland). Each sample was tested in four biological replicates.

### Quantification and statistical analysis

Quantification and statistical analyses used in this study are described in the relevant sections of the STAR Methods and figure legends. The number of animals or treatments is indicated as n. Data are presented as means ± standard error (SE). All statistical analyses were performed using Microsoft Excel, and statistical significance was determined using a two-tailed Student’s t test.
