# Supplementary material for: Haplotype-resolved genomics identifies cyp19a1a as a candidate master sex-determining gene in golden trevally (Gnathanodon speciosus)
Source: iScience. 2025 Sep 2;28(10):113493. doi: 10.1016/j.isci.2025.113493 (PMC12475581; doi:10.1016/j.isci.2025.113493)
Supplement: Document S1. Figures S1–S14 and Tables S1–S7 [file mmc1.pdf]

iScience, Volume *28*

Supplemental information

Haplotype-resolved genomics identifies *cyp19a1a* as a candidate master sex-determining gene in golden trevally (*Gnathanodon speciosus*)

Bin Fan, Jiamin Guo, Caixia Lei, Sen Yang, Zining Meng, Junyao Peng, Yongjian Yang, Yubang Shen, Yuanyou Li, and Le Wang

Figure S1 Estimation of genome size and heterozygosity from Illumina paired-end sequencing reads. Genome size was estimated to be approximately 562.6 Mb using a k-mer value of 17. The genome-wide heterozygosity was determined to be 0.452%

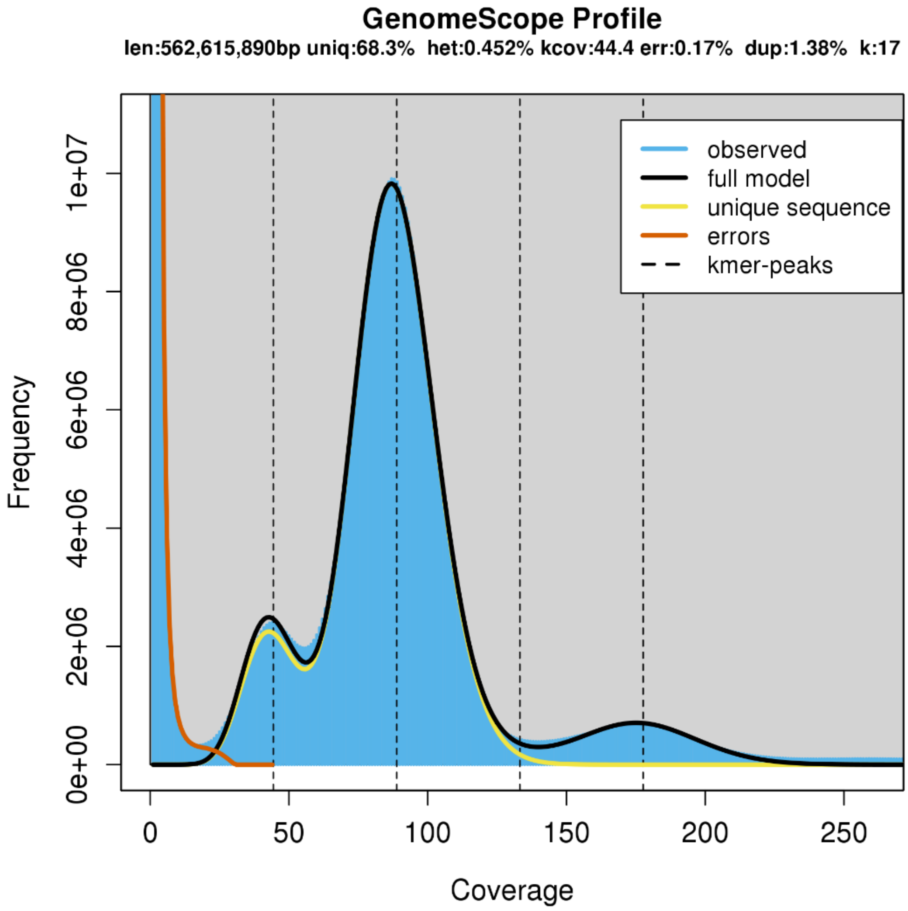

Figure S2 Sequencing read depth distribution across 60 resequenced samples.

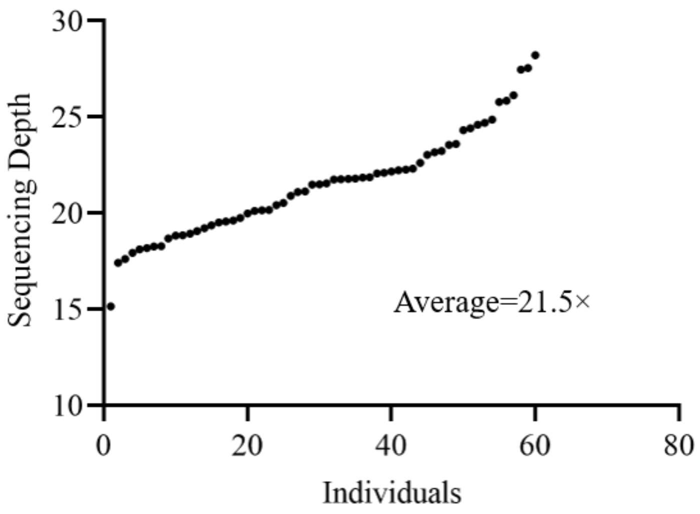

Figure S3 Population structure analysis of 30 females and 30 males conducted through principal component analysis (PCA).

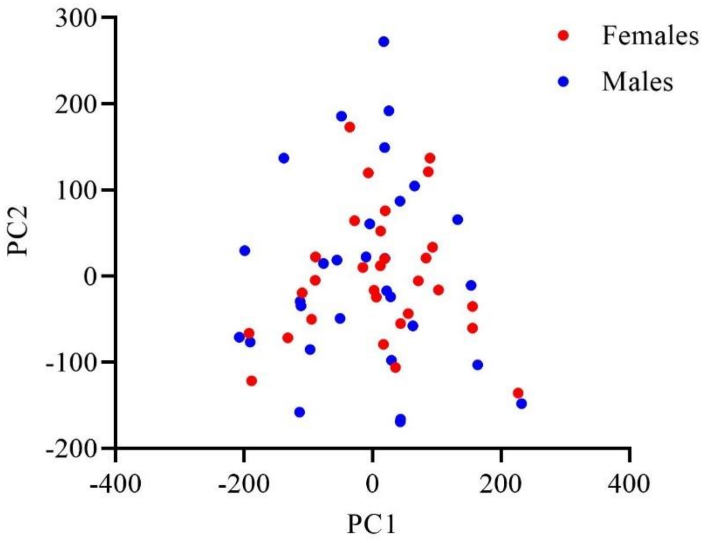

Figure S4 A quantile-quantile (QQ) plot illustrating the comparison between observed and expected *P*-values of the SNPs assessed in the whole-genome-wide association test.

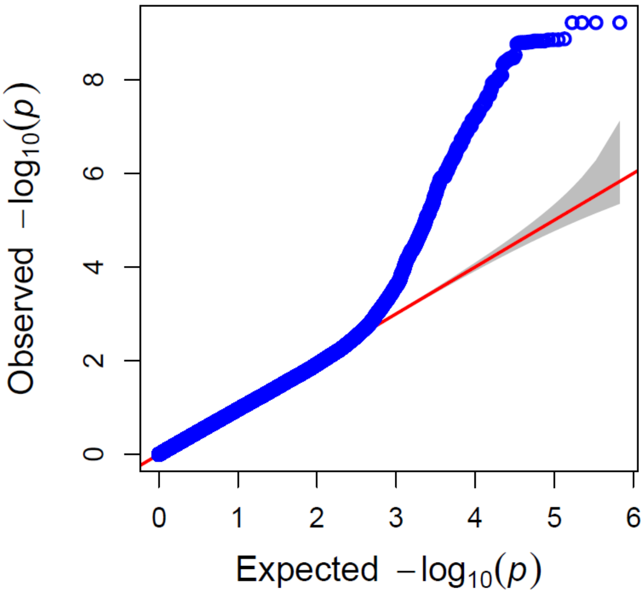

Figure S5 Gonadal differentiation between ZW and ZZ genotypes. **A**. No visible gonadal differentiation is observed between ZW and ZZ genotypes at 30 days post-fertilization (dpf). **B**. Differentially expressed genes (DEGs) in trunk samples containing gonads between ZW and ZZ genotypes. Only *cyp19a1a* exhibited female-biased expression with Log_2(fold change) > 1. **C**. Relative expression of *cyp19a1a* in trunk samples between ZW and ZZ genotypes, quantified by quantitative real-time PCR (qPCR). N = 3 per genotype and data are represented as mean +/- SE. ***, $P < 0.001$ (two-tailed Student's *t*-test).

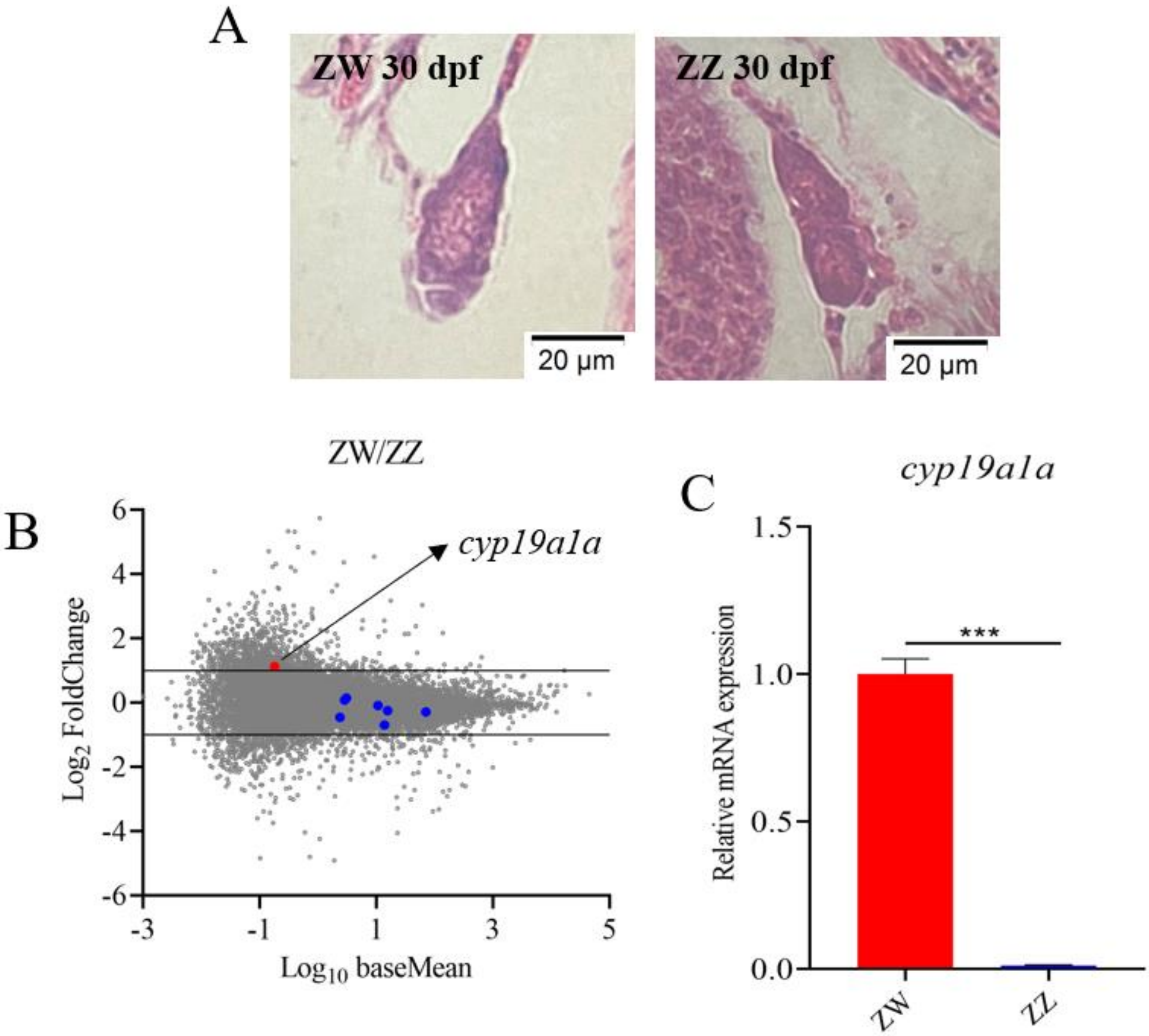

Figure S6 The predicted coding sequences of the W-linked *cyp19a1a* transcript. Coding sequences are highlighted, and conserved intron splicing sites (GT-AG) are underlined.

>cyp19a1W

ATGGCTCTGATCCCCGCCTGCGACCTGCCGCTGACTCCGGTCGGCCTGGACGGGATTCTG
ACCGATGTGGTCACCGCGTCCGCGAACGCCACCGCGGTGCAGCCGCCAGCTGTCTCCGTG
GCAACCAGGACTCTCATCCTGCTGGTCTGTCTGCTGCTGGTCGCCTGGAGCCACACGGAC
AGGAAGACCGTCCCAGGTGGGTTCGTGCAACTTTCAACACGATCACGAAACAATTTTAA
CCCTGTGACCTGAGGCAGGAACCTTCACCTGAGCTCTGAGTCTGTATCGTAACCCATTAC
AGGAGGTCACACGTCATACCTGATGCCTCACAGTCTCTGTATGTGCCGTCCTCCGCCTCC
GCAGGTCCGTCCTTCTGTCTGGGTCTGGGGCCACTTCTCTCCTACATGAGGTTCATCTGGA
CTGGAATAGGAACAGCCAGTAACTACTACAACAACAAGTACGGAGACATAGCGAGAGT
GTGGATCAACGGAGAGGAGACGCTCATCATCAGCAGGTCGGTTTTCTCTGGATCTGTTAG
ACTGAGCGGAAATCTATAATCTGTACTTTATTCATTCATTCATTCATTCATTCATGTTCCC
ACTGTGAAGGGCGTCGGCCATGTATCACGTGTTGAAGAACGGAAACTACACGTCACGTT
TTGGAAGCAAACAGGGACTGAGCTGCATCGGTATGAACGAGAGAGGAATCATCTTCAAC
AACAACGTCAGTCTGTGGAAGAAGATGAGAAGCTACTTCAGTAAAGGTAAAACCCCTCC
GTCACACACTCGGCCCTCAGACTCTGCACACCTGACGAGTCTGACCCAGAGAACTGTCCC
AGCTCTGACAGGTCCAGGTCTGCAGAACACCCTGCAGGTCTGCGTCTCCTCCACTCAGAC
TCACCTGGACGAGCTGGACAGTCTGAGTCAGGTGGACGTCCTCAGTCTGCTGCGCTGCAC
CGTGGTCGACATCTCCAACAGGCTCTTCCTGGACGTACCTCTGGACGGTGAGAGAGGAC
AGGAGTCTGTTTGACTGGTGCAAACACATGTGAGGACACTGTGTGACCTGTGTGTGTGTG
TGTGTGTGTGTGTGCAGAGAAGGAGCTGCTGGTGAAGATCCAGAAGTATTTTGACACGT
GGCAGAGCGTCCTCATCAAACCTGACGTCTACTTCAAGTTTGACTGGATTCGTCAGAAGC
ACACGACGGCAGCGTGAGTTCACCGTAACGCCTCGCTTCGTCCGTGTTGTCGAGCTTCTT
CCTGCATCAGCCCGCCTTACGGTTTGTACCTGTGTGTGTGTGTGTCAGTCAGGAGCTGCA
GGACGCCATCCAGAGCCTCGTGCAGCAGAAGAGCCGGGAGGTGGAGCAGGCGGAGAAA
CTGGACGACATCAACTTCACCACTGAGCTCATATTTGCACAGGTGAGCCTGAGAATCAAA
CTGTGATGTCACAAACGCTCCGATGGCGAAACACCCGTTCACACGCGTGTGTGCGCTTCC
AGAACCACGGCGAGCTGTCTGCTGACGACGTGGTGCAGTGTGTGTTGGAGATGGTGATC
GCAGCGCCGGACACTCTGTCCATCAGCCTCTTCTTCATGCTGCTGCTGCTCAAACAGAAT
CCAGATGTGGAGCTGCAGCTGCTGCAGGAGATCGACACTGTCGTAGGTGAGAAACTGGA
CCTGACCGCGGTGACATCATCAGTCGTACCGGTCATCATCATCAGCCAGTATCAGCTCGT
CTACACACGTCACATGTGGATTTGACATGTGGACATTGTGTGTAATAATTCAGCCTATTT
CACTCTGTTGTCGTGTGTGTGTGTGTGTGTGTGTGTGTGTGTGTGTGTGTGTGTGTGTGTG
TGTGTGTTGTTAACGGACCGTACCTAATAAAGTGACAGTGCTGCTGGTTCGTCTCCCTCC
AGGTGAGAAGCAGCTGCAGAACGGAGACCTTCAGAAGCTGCAGGTGATGGAGAGCTTCA
TCAACGAGTGTCTGCGTTTCCACCCCGTGGTCGACATCACCATGCGACGGGCCCTTTCTG
ATGACATCATCGACGGCTACAGGGTCCCAAAGGGAACAAACATCATCCTCAACACAGGC
CGCATGCACCGCACAGAGTTCTTCTGCAGACCCAACGAGTTCAGTCTGGAAAACTTTGAA
AAACATGTGAGTTTGCTCTTTTTCTTCTTCTACAAAAAGATGAAATAGCACCTGTCTCACA
CCTGTCTCACCCCTCCTTCCTCCCTCCGCCAGGCTCCTCGCCGTTACTTCCAGCCGTTCGG
GTCGGGCCCCCGGTCCTGTGTCGGGAAGCACGTCGCCATGGTGATGATGAAGTCCATCCT
GGTGACGCTGCTGTCTCAGTTCTCAGTGTGTCTCCATCGGGGTCTGACCCTGGACGACCT
CCCGCAGACCAACAACCTGTCCCAGCAGCCGGTGGAGCAGCAGCAGGAGGAGCAGCAG
CTCAGCATGAGCTTCCTGCGCCGACACAGAGGAAGCTGGAACACACACACACACACATA
A

Figure S7 The predicted amino acid sequences derived from the W-linked *cyp19a1a* transcript.

>cyp19a1W_protein

MALIPACDLPLTPVGLDGILTDVVTASANATAVQPPAVSVATRTLILLVCLLLVAWSHTDRK
TVPGPSFCLGLGPLLSYMRFIWTGIGTASNYYNNKYGDIARVWINGEETLIISRASAMYHVLK
NGNYTSRFGSKQGLSCIGMNERGIIFNNNVSLWKKMRSYFSKALTGPGLQNTLQVCVSSTQT
HLDELDSLSQVDVLSLLRCTVVDISNRLFLDVPLDEKELLVKIQKYFDTWQSVLIKPDVYFKF
DWIRQKHTTAAQELQDAIQSLVQQKSREVEQAEKLDDINFTTELIFAQNHGELSADDVVQCV
LEMVIAAPDTLSISLFFMLLLLKQNPDVELQLLQEIDTVVGEKQLQNGDLQKLQVMESFINEC
LRFHPVVDITMRRALSDDIIDGYRVPKGTNIILNTGRMHRTEFFCRPNEFSLENFEKHAPRRYF
QPFGSGPRSCVGKHVAMVMMKSILVTLLSQFSVCLHRGLTLDDLPQTNNLSQQPVEQQQEE
QQLSMSFLRRHRGSWNTHTHT

Figure S8 The predicted coding sequences of the Z-linked *cyp19a1a* transcript. Coding sequences are highlighted, and conserved intron splicing sites (GT-AG) are underlined.

>cyp19a1Z

```
TGCGACGTGTTGATGAACCAACAGCTGCACGTGTCGGAGCTTGCCTGCTCCTGGTGCCGA
CTGAGCGGACTTTCATGCGGACAGCGAGGCTCGCGGAGCTCCGAGCTGCAGACGGTGGA
GGAGGTGAGACGGGTTCCTGAAGGTTCCTGTGTCACAGACGAACCCTCTGATCCTCAGA
GGACAGAGCACCGAAGTGTGCTGTTGCACGTGTGTGTGTGTGTGTGTGTGTGTGTGAGGG
AAGCAGCTGTTGTGCAGATGTTCTTACCTGGAGAGAGGTCTCCGTCACTCTGCTCTCCGG
AGTCTGAATCCATCTTCTGCTCCTGGAGGACACTCGGAGGCTGCAGAAGCTCTCTGATCC
CTCGCTCTTCTTCTCCTCCTCCTCCTCTCCAGAGACGTCCTCTGGTTTCTTCAGCCACA
GGAGAAAAACTAAGCGATATTTCTTGTGTTTCTTCTCGTCTCCAGTTGGAGCTGAACTTCC
CTCCCAGCAGCTGTGAGAGGAGGAATGTTAACTGGGCTGATCTGCTGAGCTGTCCTCCTC
CTCCTCCTCTCTTCTGTCTTTCTCTCTCTCTCTCTCTCTCTTTTTTTTGCTCTCCTTCCCCT
TCGCTCTCCTCTCTTCTCCTGACTCCCTCAGTTTCTCTCTCATCTACTTTCTCTGATGCTCC
CTCCCCTCGTTCACTCTCGGTCCGTCCCCCTCTCTGTTCCTCCCTCCCTCGACCCTCCTCCT
CCTCCTCCTGTCACACTCGGCCTCTTCACGCTCTCTCTCTCTCCACTCTCACTCTTTCTCTA
CCTCTTTCTGTATGCTGTAAGTGCCGTCTCTTCCTCCCTCCCTCCCTCTCTCCCTCCCTCTC
TCAGCCTCTGAGTGCTGCCTGTGTTTTAGCAGAGCAGTGGGAGAGAGTCATTCATGGCGA
CTTGAAATTCATCTCCACCAGTTAAAAATCCCATGACCACTTGTTACTGGCTGTTACAGA
ACCAGTCCATCTCTCTCTCTCTCCCTCTCTCTCCCTCTCTCTCTCTGTGGCTGCGATGCATT
CGCTGACTGACCGCAGTAAAGTGTTGTATGTCCTTTAAGTGAGGTAGAAAAGTTGTGTTG
TTGGAGTTTCATTAGATGATAAAAATAAAGCAGCAGCAAAGTAAAAATAGACAAAACAT
CGTCGTCTTCACAATAAGAGATGTGTTCTCTCTGGAGAATCACACACACACACACATCGG
TGCTGTAAGGCCGGTAACTTTACACTGATAAACAAACCGCAAAGTTAATGAGGAGATTTT
TAACAAGTGAAGATTCACTAAAGTCACTAAAATCAGAGTTTATACTCAAACTAGACTCA
GTAAAGTTAAAGCTGAAGGTGGTCAGTGCAGCTCAGCAGAGTGAGGTTACACTGCTGCC
GTCTGTGGATTTATGCCAATTATATGTTTGTTTTATTGTTTTTTTATCTTATTTTGATAGTT
TCATTTTTCCACTTTTCCATCGGGAGTCTGTTTGTAACCAAAGAATTTCAGGATCAATAAA
GTGATTCTGATAAAACATGACGTTTAGAATCAGAATCAATAACAGCTTCTTTAGTGATTT
GCTGGTTTGTAGTAAATTAATTTCTCCTCCCACTTAAAGGAGCGAATGGTAATGACCGTG
TTACCTGATGGTTTCCCACACCTGAAAACTACAGAACACAGGAATTCTGGGAAATGGTGT
TGTAGGAAGAAAACAGAGAGACTGGTGTTGTATGAACTGAGGTCAGGGTGGGTTTCTCT
TTTAATTAACCGCAGTGTGAGGCGGAGGTGGGGGGTCGGGGGGGGGCAGGTGTGTGTC
CAGTCGGTGGTCCGACAACAGTTGCAGGCGATTCTCCCTCCCAGGTGTGGAGCTGACCGT
TGCTCAGCAGGAAACACTGGGCACCAGATGGCGCCGTCCTGTCTGCCCACTGCCCAGGG
CGCCTTTATCTTTGACTCTGTGTGTGTGTGTGTGTGTGTGTGTGTGTGTGTGTGTGTGTGC
ACTGATTCTCCAACATTGCCCAGACAGAGCAGACACAAGCTGAGCTCCTCTGATCCTCAC
ACTGAACCTCTGCACGTCTTTGACCTGGTGTGAAACGACTTGTTTTTGTAAAGTCAGAA
GTTATGAAAACAGAAACAGAAATATTCTTCTGAAGCTCTGGCCTTCAGCGTGTGAGCGTC
AGCCTCTGGTGTTTTGGCCGAGTCTCAGTTTCTGATCCAGTCACCATCGTTTGAGTGTGTG
TGTGTGTCAGTTGTGTTGTTGTATAAGACTCTGTCAGATCTACTGTGTCTCTGCTGGAGAG
CACGACTCGCACAGAGTTCACAATGTAAAAACAAAGTCTACTTGATTAATACAGCACATT
TATCAGGTCTTACAGGACTGGATCAGAGTACTGGACTGGATCAGAGTACTGGACTGGAT
CAGAGTACTGGACTGGATCAGAGTACTGGACTTATCGCTGGAGTAAATCTCCTTCCTGTC
CGCTCCAATGATCCGTCCTCGCTGACTGTGTTTGAAAATGTTTTGGATTCCTTAACTTTCC
CAAACTGGTGTTACAGACTGAAGAGACAGAGACGGCTCACGCTGTAGAGAGAGGAGGA
GAAGGAGGAGAAGAAGGAGGAGGAGGAAGAGGAGGAGGAGGAGGAGGAGGAGG
AGGAAGAGGAGGAGGAGGAGGAGGAGGAGGAGGAGGAGGAGGAGGAGGAGGAGAGC
AGCCTCCTGTAATGATGTGCAGCACCGCTCTCCTCTGATGTTCAATGAGAAACAAATGAG
CTGACTTTTCTGAGCGGCCACATGAAAAGACTGTGGAGTTAGAGTTCGCCTTTGATCCGG
TGCCTTCACTGGCACACGGTCTTGGATGGGTCTTCACAGAGACCTGGTCTGATCACAGAG
ACCAGGTCTGATCACAGAGACCCGGTCTGTCCTCAGAGACCCGGTCTGATCACAGAGAC
CCGGTCTGATCACAGAGACCTGATCTGTCCTCAGAGACCTGATCTGTCCTCAGAGACCAG
GTCTGATCACAGAGACCTGGTCTGTCCACAGAGGTCTGATCACAGAGACCCGGTCTGTCC
ACAGAGACCTGGTCTGTCCTCAGAGACCCGGTCTGTCCACAGAGACCGGTCTGTCCTCAG
AGACCTGGTCTGTCCTCAGAGACCCGGTCTGATCACAGAGACCTGATCTGTCCTCAGAGA
CCAGGTCTGATCACAGAGACCTGGTCTGTCCTCAGAGGTCTGATCACAGAGACCTGGTCT
GTCCACAGAGGTCTGATCACAGAGACCTGGTCTGTCCACAGAGGTCTGATCACAGAGAC
CCGGTCTGTCCACAGAGACCAGGTCTGTCCTCAGAGACCTGGTCTGTTCACATAGACCTG
GTCTGTCCTCAGACGTCTGATCACAGAGACCAGGTCTGATCACAGAGACCTGGTCTGATC
ACAGAGACCAGGTCTGATCACAGAGACCAGGTCTGATCACAGAGACCTGGTCTGATCAC
AGAGGTCTGATCACAGAGACCTGGTCTGTCCTCAGAGGTCTGATCACAGAGGTCTGATCA
CAGAGACCTGGTCCAGAGTCCGGCTTCATATTTGGTTCTGTTGTCTCGTGATCGGAAGAA
GCGTCAGGAGCCTGAGACAGAAACGGAGTCAGAGCTCCTGACTCGCTCACGTTGACACA
TCGCCGCTTTAGCCTCTCTGCTAGGTGTTTCCATGGCAACCACTGCTCTGTTTTTAATGAA
GCTATAATCCAGCAGGGGATGGAAACAGACACAATATGGCCACTGGCATTTCAGAGTTT
TAGCACAATATAAAGTGTGTGTGTGTGTGTGTGTGTGTGTGTGTGTGTGTGTGTGAGCAG
CACATAATCAATGAGCTGTCAATCACAGTGCGAGGCAGGACAGTCTAAAGTCGTAGCTG
GAGGCTCCTCTTCTCTTACGTCGGAGTTATTTTGGAGGCCGTCTGGCGTCCTGCCTCTGTA
AATCTGTCATGCTGCAATAGGAATAAATGGAATGAGACGGAAAATGTAGATCATGTGAC
CGACAGAAACCACATATGAAGAACCAGAGAGAGGCTGAAGCAGCAGCAGATCGTTCAG
CTCCAGTCGTTACAGCGAAGTGGGTTCAGCTGCTCTGAGAGTCTGCAGACGACCTCCGAC
CCCGAGCCTGGAGCAAGAGCACCGTTCAGCTCCCCGTTAAAAACATTTCATAATCTGACT
CACACACACACACACACACACACACACACACTGTAACACACCGCAGACTCACTGTAGACTT
GAAGTGAAAGTTACCACAGATCTGTTTTGCAGATGCCTGATAACCCACAAGTCTGGAGCC
AGAGGAGACGGAGAGAGGAGGAGGAGGAGGAGGAGGAGGAGACGGACGGACGGG
AGTTCACGGAGACACACAGGAGACGGAGACACGCGAGTTTATACTGAGACGACAGAGA
GACGAGAGACGACGGGGGCTGTCGGTCCGTCTGAGGTGCAGCGTGAAGGACAGACAGA
TGGATGTGATTGAAGATGACAGGCTGAGGTGATAATTAAAGCTGGTTCTGCCCTGAAGCT
GCTACACACACACACACACACACACACACTTTTTGCTCTAACACAAACAGTTATTGATTA
GTTATAGATCAATACATGTGTGTGTTCCTGTCGTGATGTTGATCAGGAGCCGGAGATGAA
GGTCTGGTGCCAGCAAGCTTCACAGAGACGATGGAGATTATTGTGACGAGCAGCGTTTT
ACTGCCAGGTACAGTGGTGTGTGTGTGTGTGTGTGTGTTTGTGTGTGTGTGTGTGTGTG
TGTGTGTGTGTGTGTGTCAGCGTCAGCCTGTTCTTTGCAGGTTTCTTGGCTTTCAGTGGGT
GAATCACTATATCTCATTAACCTCAGCCTCATTCCTGTCCTCCATCCAGATGGAAACCATC
TCACTACACACACACACACACACACACACACACACACACACACACACACACAGACCAACAC
TTGGAAAATAACAATTTTAGAGGAAAACACACTAATGACCAAACTTCAGGCTCAATTTG
AACTTTAATACAGCACAGTGTGTGTGTGCGTGTGTGTGTGTGTGTGTGTGTGTGTGTGT
GTGTGTGTGTGTGTGTGTGTGTGTGTGTTGCTGAGGTGGGGAAATGACACTAACACATTTCT
TCCTGTTGCATCACTGACAGGAGTTGATGTTTGTCGTCTCTGGAGAATCTCTCACCTGGA
AACTGTCTTCACCTGCGTTTCCTCTTCTTCTTCCTCTTCTTCTTCTTCTTCTTCTTCTTC
TTCACCTCCTCCATCTCTGTTAGCTTTGAGTCTTTGTCATCTGTGCTCTCTCACTTCCATCC
TCTGAGTCCCAGAGCTGAGGAAGTGTGTGTAAAACAAACTGTATTTTTATATGTGTGTTG
CAGCGCCCTCTCCTGACCAGACAGTGTCAGTGCAAAAGCCCCCACAGCCAACACACACT
CACACACACACACAGGTCATCCTGCTCTTTTTTCTCCAGTTTGCTTCAAATAAACACAAA
AGTTTAAGTTTTCAATAAATTCAGTTTTATTATTTATTTATATATATATAAAATTATATATTTT
TATTATTCTATTATAATAAAAATACATTTTTTTCCACCTTTCAAACACAAAATGCAGCAAA
AAGTGCAAAAAACATTTCATGTGTAAAATGAGAAGGCACCAGAAACACACACACACACA
CACACACATATCTTTACATGGACGTCAGTGTGAGTGTGTGTGAACCTGTCGCTCAGTTAG
ATCCTGTAGAAACACACTTTTTAATACTAAATATACCAAATAAATACTTTGAATACTAAC
AGTAACAGACACAGCAGGAACGATGCGACGGACTCGTGTGTGTTTATGTGTGTGTGTGT
GTGTTCCAGCTTCCTCTGTGTCGGCGCAGGAAGCTCATGCTGAGCTGCTGCTCCTCCTGCT
GCTGCTCCACCGGCTGCTGGGACAGGTTGTTGGTCTGCGGGAGGTCGTCCAGGGTCAGAC
```

continued

```
CCCGATGGAGACACACTGAGAACTGAGACAGCAGCGTCACCAGGATGGACTTCATCATC
ACCATGGCGACGTGCTTCCCGACACAGGACCGGGGGCCCGACCCGAACGGCTGGAAGTA
ACGGCGAGGAGCCTGGCGGAGGGAGGAAGGAGGGGTGAGACAGGTGTGAGACAGGTGC
TATTTCATCTTTTTGTAGAAGAAGAAAAAGAGCAAACTCACATGTTTTTCAAAGTTTTCC
AGACTGAACTCGTTGGGTCTGCAGAAGAACTCTGTGCGGTGCATGCGGCCTGTGTTGAGG
ATGATGTTTGTTCCCTTTGGGACCCTGTAGCCGTCGATGATGTCATCAGAAAGGGCCCGT
CGCATGGTGATGTCGACCACGGGGTGGAAACGCAGACACTCGTTGATGAAGCTCTCCAT
CACCTGCAGCTTCTGAAGGTCTCCGTTCTGCAGCTGCTTCTCACCTGGAGGGAGACGAAC
CAGCAGCACTGTCACTTTATTAGGTACGGTCCGTTAACAACACACACACACACACACACA
CACACACACACACACACACACACACACACACACACGACAACAGAGTGAAATAGGCTGA
ATTATTACACACAATGTCCACATGTCAAATCCACATGTGACGTGTGTAGACGAGCTGATA
CTGGCTGATGATGATGACCGGTACGACTGATGATGTCACCGCGGTCAGGTCCAGTTTCTC
ACCTACGACAGTGTCGATCTCCTGCAGCAGCTGCAGCTCCACATCTGGATTCTGTTTGAG
CAGCAGCAGCATGAAGAAGAGGCTGATGGACAGAGTGTCCGGCGCTGCGATCACCATCT
CCAACACACACTGCACCACGTCGTCAGCAGACAGCTCGCCGTGGTTCTGGAAGCGCACA
CACGCGTGTGAACGGGTGTTTCGCCATCGGAGCGTTTGTGACATCACAGTTTGATTCTCA
GGCTCACCTGTGCAAATATGAGCTCAGTGGTGAAGTTGATGTCGTCCAGTTTCTCCGCCT
GCTCCACCTCCCGGCTCTTCTGCTGCACGAGGCTCTGGATGGCGTCCTGCAGCTCCTGAC
TGACACACACACACACACAGGTACAAACCGTAAGGCGGGCTGATGCAGGAAGAAGCTCGA
CAACACGGACGAAGCGAGGCGTTACGGTGAACTCACGCTGCCGTCGTGTGCTTCTGACG
AATCCAGTCAAACTTGAAGTAGACGTCAGGTTTGATGAGGACGCTCTGCCACGTGTCAA
AATACTTCTGGATCTTCACCAGCAGCTCCTTCTCTGCACACACACACACACACACACACA
CAGGTCACACAGTGTCCTCACATGTGTTTGCACCAGTCAAACAGACTCCTGTCCTCTCTC
ACCGTCCAGAGGTACGTCCAGGAAGAGCCTGTTGGAGATGTCGACCACGGTGCAGCGCA
GCAGACTGAGGACGTCCACCTGACTCAGACTGTCCAGCTCGTCCAGGTGAGTCTGAGTG
GAGGAGACGCAGACCTGCAGGGTGTTCTGCAGACCTGGACCTGTCAGAGCTGGGACAGT
TCTCTGGGTCAGACTCGTCAGGTGTGCAGAGTCTGAGGGCCGAGTGTGTGACGGAGGGG
TTTTACCTTTACTGAAGTAGCTTCTCATCTTCTTCCACAGACTGACGTTGTTGTTGAAGAT
GATTCCTCTCTCGTTCATACCGATGCAGCTCAGTCCCTGTTTGCTTCCAAAACGTGACGTG
TAGTTTCCGTTCTTCAACACGTGATACATGGCCGACGCCCTTCACAGTGGGAACATGAAT
GAATGAATGAATGAATGAATAAAGTACAGATTATAGATTTCCGCTCAGTCTAACAGATC
CAGAGAAAACCGACCTGCTGATGATGAGCGTCTCCTCTCCGTTGATCCACACTCTCGCTA
TGTCTCCGTACTTGTTGTTGTAGTAGTTACTGGCTGTTCCTATTCCAGTCCAGATGAACCT
CATGTAGGAGAGAAGTGGCCCCAGACCCAGACAGAAGGACGGACCTGCGGAGGCGGAG
GACGGCACATACAGAGACTGTGAGGCATCAGGTATGACGTGTGACCTCCTGTAATGGGT
TACGATACAGACTCAGAGCTCAGGTGAAGGTTCCTGCCTCAGGTCACAGGGTTAAAATT
GTTTCGTGATCGTGTTGAAAGTTGCACGAACCCACCTGGGACGGTCTTCCTGTCCGTGTG
GCTCCAGGCGACCAGCAGCAGACAGACCAGCAGGATGAGAGTCCTGGTTGCCACGGAGA
CAGCTGGCGGCTGCACCGCGGTGGCGTTCGCGGACGCGGAGACCACATCGGTCAGAATC
CCGTCCAGGCCGACCGGAGTCAGCGGCAGGTCGCAGGCGGGGATCAGAGCCATGGCGA
GGGTGGGACGAGCTCCGCTGCACCAACCGGAGGTGGAGGCTGCCTCAGCACCAGGACTC
GGCTTTATATCAGCAGACGGAGCTGGTCCTCAGCCAGGTCAGGGTAACACACACGCCAC
AACAAAGAGGCTCAGAGGTAAACAACACACTGGCAGCCTTGAGGCTCTGGGCGTGAGTC
GGATCCTTGAGCAGAGAGGGTTCAGTGCTGTGGAGGAGGAGGAGGACGATCTGTTATCT
CCTGTTTGACTCTCTGACTCTGGAGCTGCTTTGATCATCGAACGTCTTTCAGCAGCAACAT
GAAAACACTTCCTTTCGTTATTATAACTCCGTGTCATGACAAGCGAAATTAACAGGAAAC
ATTAAAACGTCTGTTTGATGTGGTTGTCGGTTCATGACGTTGTTTCCACCAGTAGATGGA
GAAGCAGAGCTGCACCGAACACACATGTCATCATTGCTCTGAGTGAGGTGATCCAAGGA
CAGCACCTGCACATGGACCGTCTCCTCTTCAAACAGGTCCTTACCAATAAAAAGACAGTG
AGAGTACAGAACAAATCTGTTAATGTCTACACACAGGCCCAGTCCTGTGTGTAGACATTA
TTATTATTTATGAAGTTATTTGGCTTTTATTTTGATAACTCTGCCAATAAACTGGTGGTTC
AGTCAGCTTCATTCATCAGAATCAGTGTGAACAGAAACAGATGAGGATGATGATGATGA
GCAGAAAAATCACATCATTATTACATTTATTACATAAATTCATTGTTATATAAGGAGCTG
AAAGTCAAACAGGGCCTGTAGCATGAACAGCTGCTGCGTCTTTAATAACATGCTCTAATC
TCACAGACTTTTCTCATGTTTGTTTCTTTGTTGAATATAAAATATTTCTATCTGTTCAAGAT
TCAAACAACTTTATTTATCCCCAAAAGTCAGACAATTCCCAGTCAGTCCGTCAGTAAAAC
AGCAGCATAAGTATGTGATAATTTTATTTTATTTTATCCCCCATGCCCCTGACTCTTTGCC
CCGACCCCCCCCCCCACCCTCTTTGGATAATGGACTCTGTGAATTCACTCTCGCCCACTCT
GGGCTGCAGGAATGCGAGGCGTTCAAGCGTGCGACAGTGCGAGGCCTGTGTCACGGCTC
AGATTGTTCTGTGTGATTAAACGGAGACAAACGCTGCGTTTGAGCCGCGGAGCAGGAGT
TAGAGTAACGTGGTCGAACCGCAGCTTTGTCAGAGAGAGGAGGAGAAGAAGAAATGTC
AGAGCATGAATCCACTGACATCATCATCGTCTCTGTGTCTCCACGTGTTGGATAGAAACA
CAGAGGAGTCAGAACTTCACCTTGTGTTTATCCGATGACATCATGACTTATATGCTTACA
TCATCACCTCATTGGAAACTGTCTCTCCTTCTCTTTCAGTTGAAGTCAAACTCACCCGATG
CAACAAACACCGTGGTTTAATCAAACAGTGAGCGTGTGAGGCGTGTCAGACGGACACGG
CGTAGAGGAGGACGAGGCTGTGTCGTGTCATTGTCATTGTTGGTCTGAGGCGGAGCAGCT
GGACCTGGGTCTGAGGCGGAGAGCTGGACCTGGGTCTGAGGGGGAGAGCTGGACCTGGG
TCTGAGGCGGAGAG
```

Figure S9 Comparison of *cyp19a1aW* and *cyp19a1aZ* Sequences. **A**. Schematic representation showing that the predicted protein-coding genes from the W- and Z-linked *cyp19a1a* alleles, *cyp19a1aW* and *cyp19a1aZ*, respectively, utilize different antisense strands as transcription templates. **B**. Partial complementarity between *cyp19a1aW* and *cyp19a1aZ* transcripts. Transcripts from *cyp19a1aW* and *cyp19a1aZ* were aligned based on their genomic coordinates, with complementary homologous regions indicated by dashed lines. **C**. The relative expression of *cyp19a1aW* and *cyp19a1aZ* transcripts in ZW ovaries and ZZ testes. Allele-specific expression of *cyp19a1aW* and *cyp19a1aZ* transcripts was examined using qPCR with transcript-specific primers (n=3 per genotype and data are represented as mean +/- SE). (*P* values for two-tailed Student's *t*-test are provided).

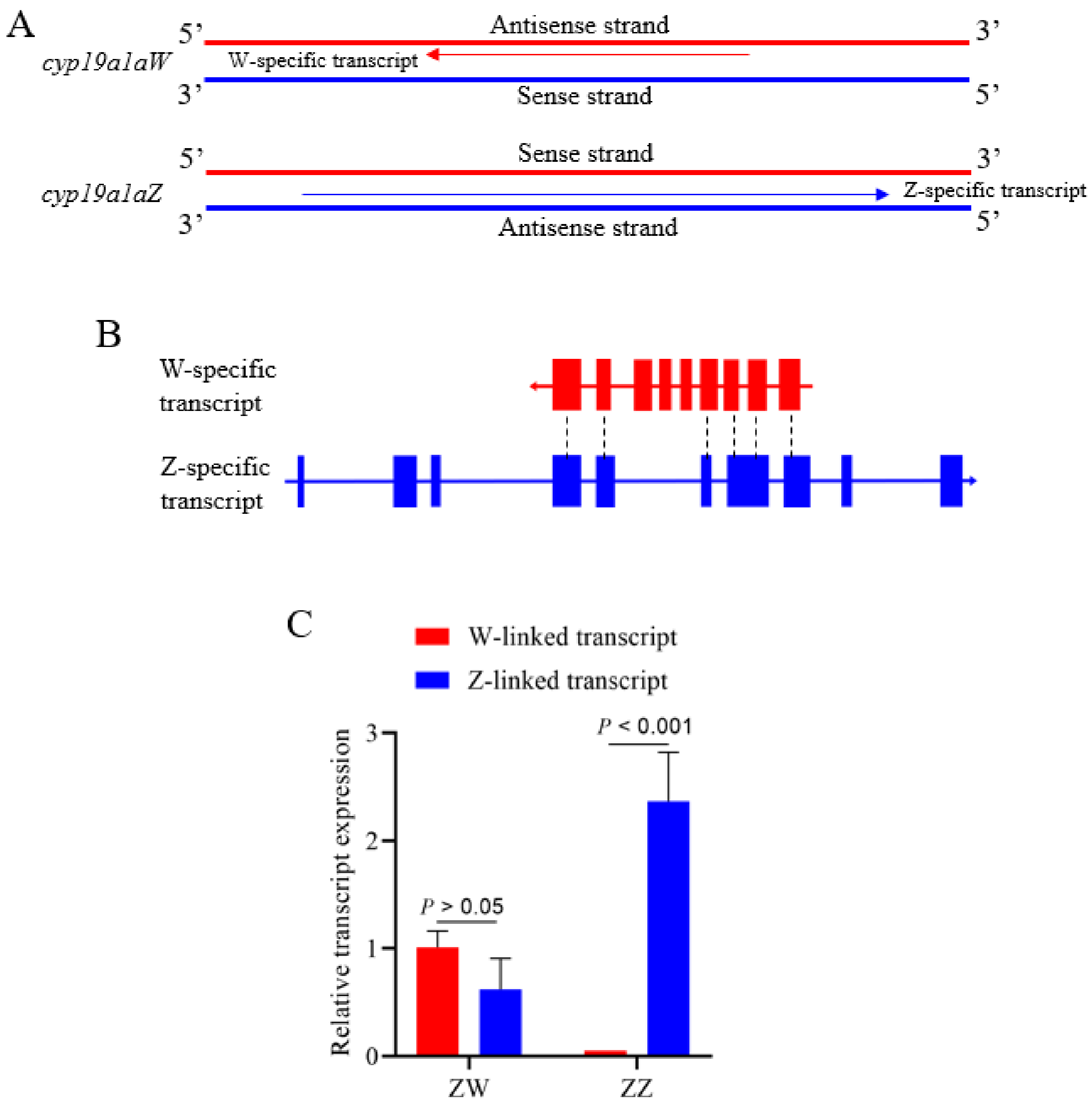

S10 Expression of *cyp19a1b* in transcriptomes of golden trevally. **A**. The expression of *cyp19a1b* in the ovary and testis, calculated in transcripts per million (TPM), as revealed by transcriptome sequencing. B. The expression of *cyp19a1a* and *cyp19a1b* in trunks at 30 days post fertilization (dpf) between ZW and ZZ genotypes revealed by transcriptome sequencing (n=3 per genotype and data are represented as mean +/- SE, with * indicating $P < 0.05$ and ns indicating not significant for two-tailed Student's *t*-test).

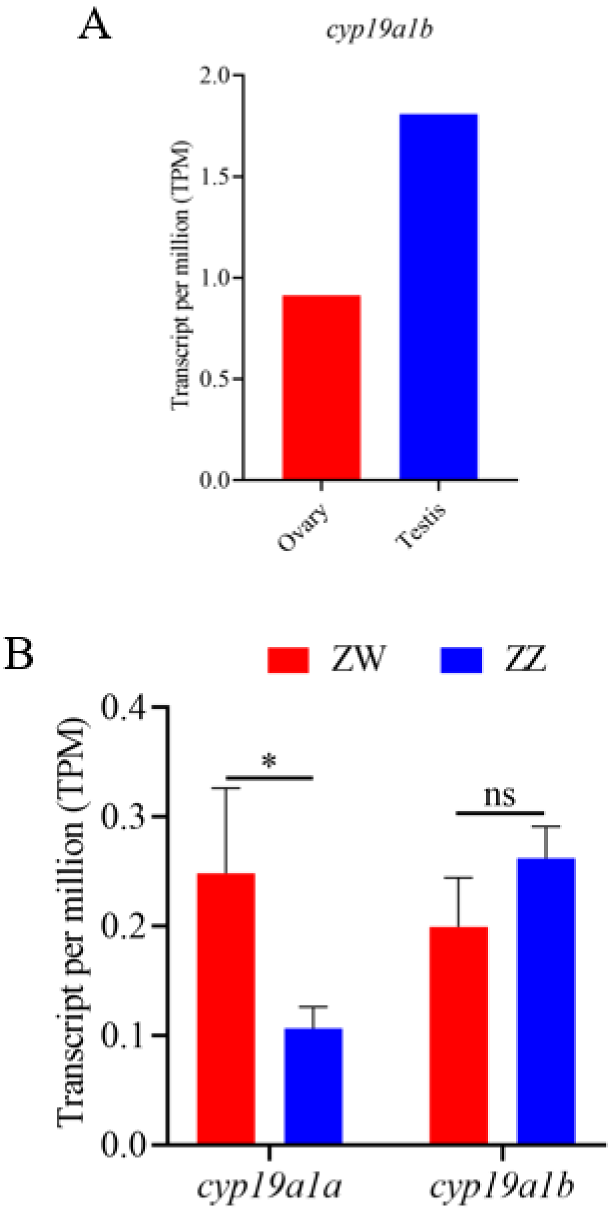

Figure S11 A highly repetitive sequence of approximately 400 bp is inserted upstream of *cyp19a1aZ*. **A**. The ~ 400 bp insertion is sex-specific across the entire mapping population. Sanger sequencing indicates that PCR artifacts in ZZ genotypes are likely stutter bands caused by DNA polymerase slippage during the amplification of highly repetitive sequences. **B**. The ~ 400 bp insertion in the *cyp19a1aZ* locus contains highly repetitive tandem repeats, with repeat motifs highlighted iteratively in two different colors.

A

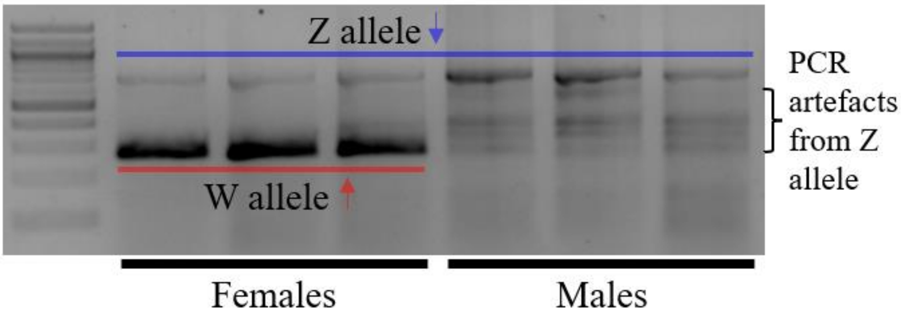

B

>Chr01: 30796439-30796877

CATTGTCATTGTTGGTCTGAGGCGGAGCAGCTGGACCTGGGTCTGAGGCGGAGAGCTGGACCTGGGT
CTGAGGGGGAGAGCTGGACCTGGGTCTGAGGCGGAGAGATGGACCTGGGTCTGAGGGGGAGAGATG
GACCTGGGTCTGAGGCGGAGAGCTGGACCTGGGTCTGAGGCGGAGAGCTGGACCTGGGTCTGAGGC
GGAGCAGCCGGACCTGGGTCTGAGGCGGAGAGCTGGACCTGGGTCTGAGGCGGAGCAGCCGGACCT
GGGTCTGAGGCGGAGAGCTGGACCTGGTCCTGAGGCGGAGAGCTGGACCTGGGTCTGAGGCGGAGC
AGCCGGACCTGGGTCTGAGGTGGAGAGCTGGACCTGGGTCTGAGGCGGAGCGGCTGGACCTGGGTC
TGAGGTGGAGAGATGGACCTGGAGAGGAGGAACATGAGGAG

Figure S12 Distribution of read depth around the sex determining region (SDR). **A**. Depth of PacBio sequencing reads from ZZ and ZW genotypes separately mapped to the Z chromosome. **B**. Depth of PacBio sequencing reads from ZZ and ZW genotypes separately mapped to the W chromosome. Sequence depth is normalized between ZW and ZZ genotypes.

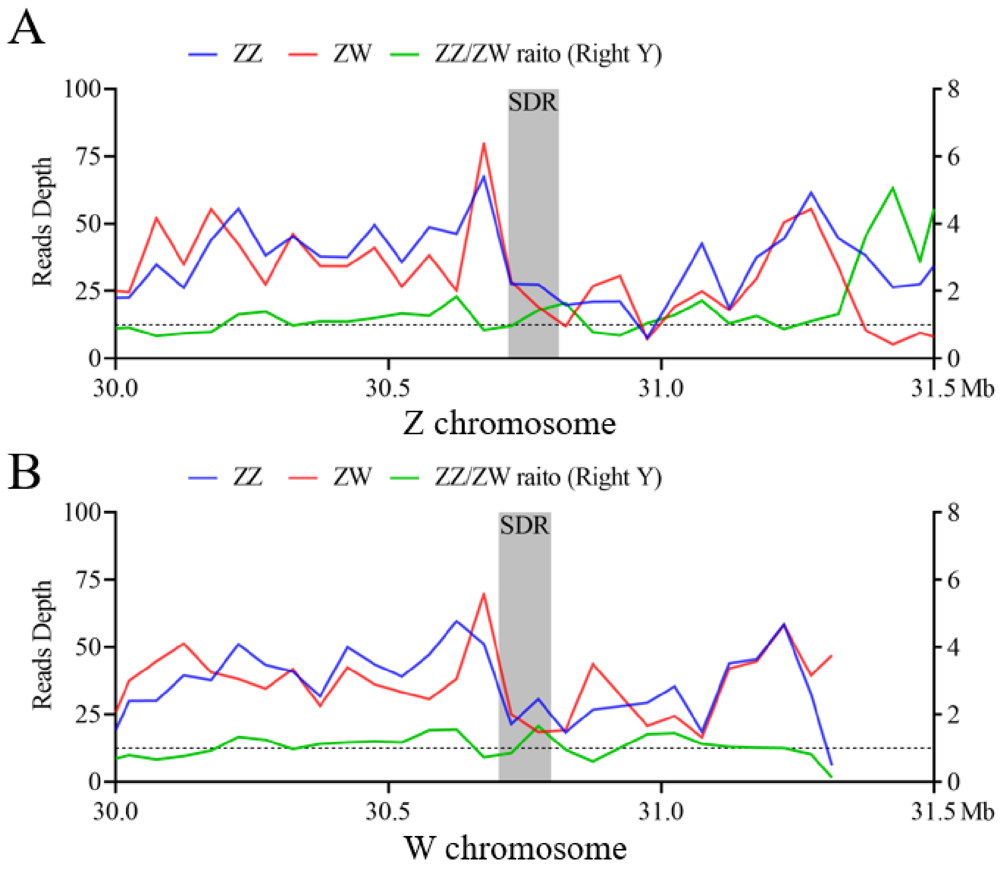

Figure S13 Distribution of repetitive elements around the sex determining region (SDR). **A**. The distribution of repetitive elements around the SDR on the W chromosome. **B**. The distribution of repetitive elements around the SDR on the Z chromosome.

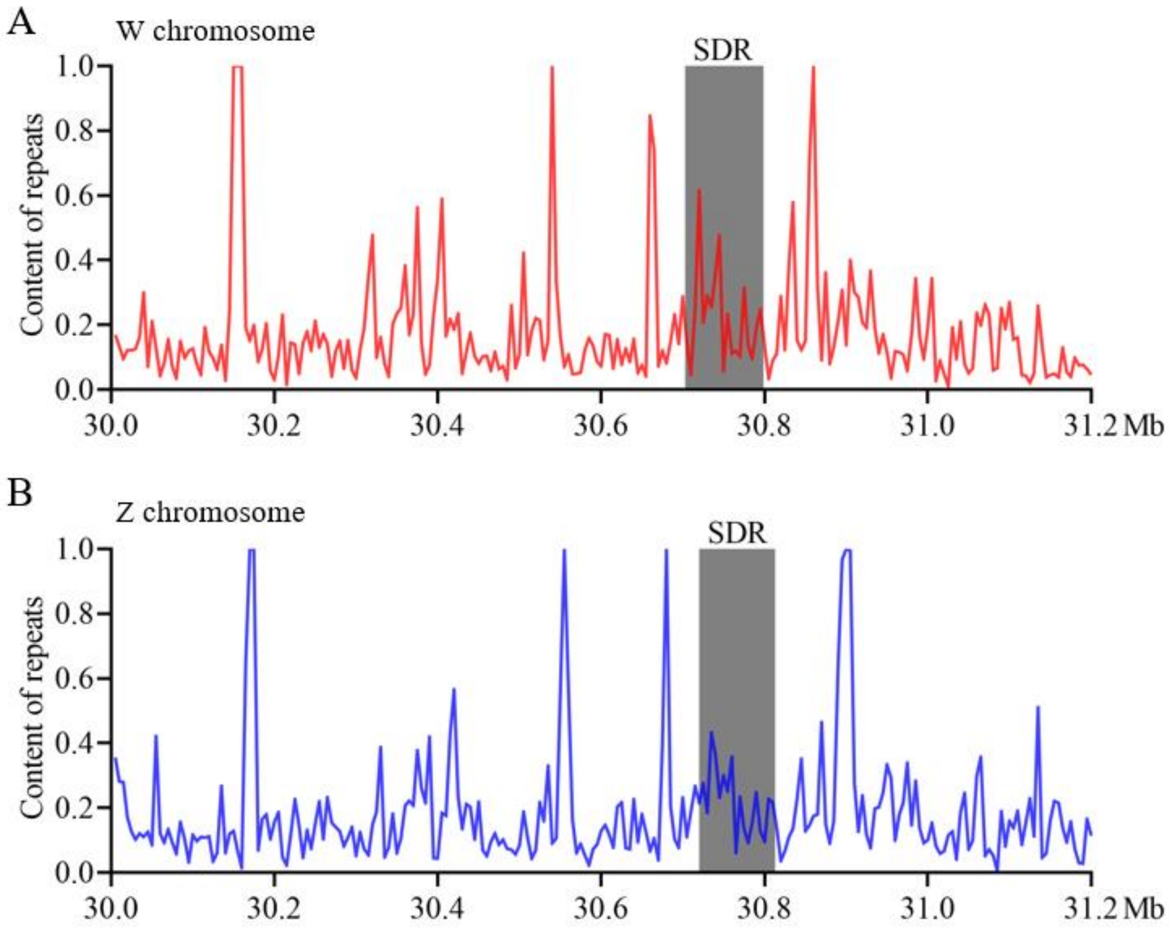

S14 Estimated population scaled recombination rate (ρ = 4Ner) along sex chromosome. The sex determining region (SDR) and putative recombination suppressed region (RSR) are indicated.

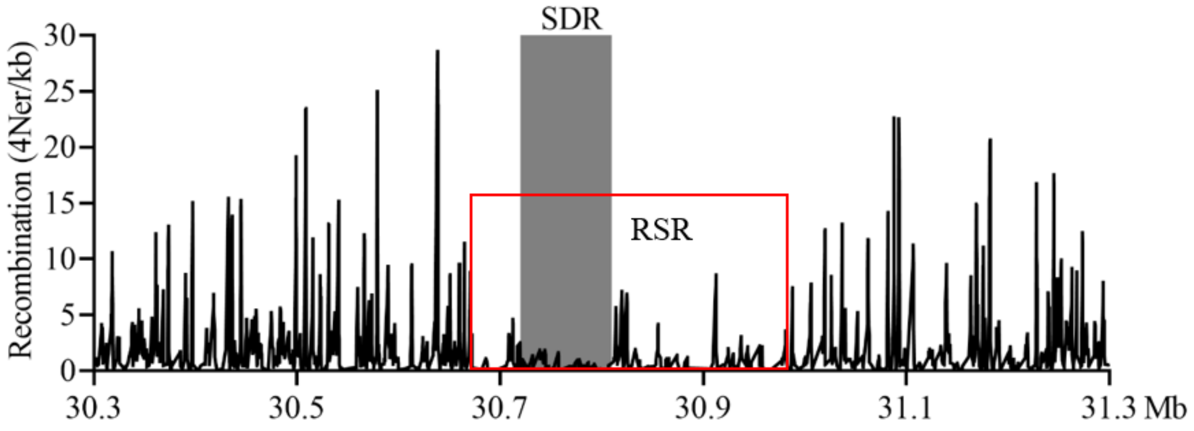

Supplementary Tables

Table S1 Samples and sequencing reads used for whole-genome sequencing and RNA sequencing.

Sample	Sequencing	Reads	Length	Annotation	Uniquely mapped
Pacbio_m	Pacbio HiFi	2.5 M	16.0 kb	Genome assembly	n.a.
Pacbio_f	Pacbio HiFi	2.2 M	16.3 kb	Genome assembly	n.a.
HiC_m	Illumina NovaSeq	396 M	2x150 bp	Genome assembly	n.a.
Genome_m	Illumina NovaSeq	197 M	2x150 bp	Genome survey and polish	n.a.
RNA_C3	Illumina NovaSeq	99 M	2x150 bp	ZZ trunk sample at 30 dpf	87.46%
RNA_C4	Illumina NovaSeq	71 M	2x150 bp	ZW trunk sample at 30 dpf	86.79%
RNA_C5	Illumina NovaSeq	70 M	2x150 bp	ZZ trunk sample at 30 dpf	85.77%
RNA_C6	Illumina NovaSeq	78 M	2x150 bp	ZW trunk sample at 30 dpf	85.32%
RNA_C7	Illumina NovaSeq	91 M	2x150 bp	ZW trunk sample at 30 dpf	84.44%
RNA_C8	Illumina NovaSeq	71 M	2x150 bp	ZZ trunk sample at 30 dpf	86.17%
Testis	Illumina NovaSeq	27 M	2x150 bp	Gonad transcriptome	90.59%
Ovary	Illumina NovaSeq	20 M	2x150 bp	Ovary transcriptome	89.67%
Liver	Illumina NovaSeq	39 M	2x150 bp	Liver transcriptome	89.35%
Eye	Illumina NovaSeq	26 M	2x150 bp	Eye transcriptome	90.17%

Table S2 Summary statistics of assembled contigs and scaffolds of the male and female genome sequences.

Genome assembly	Male (ZZ)		Female (ZW)		Female hap 1 (ZW)		Female hap 2 (ZW)	
	Contig	Scaffold	Contig	Scaffold	Contig	Scaffold	Contig	Scaffold
Number of fragments	246	64	301	30	331	33	211	32
Min fragment length	14.3 kb	14.3 kb	10.8 kb	41.2 kb	18.5 kb	39.6 kb	10.8 kb	20.0 kb
Max fragment length	27.5 Mb	31.7 Mb	27.4 Mb	31.9 Mb	27.2 Mb	31.9 Mb	27.2 Mb	31.3 Mb
N50 Length	21.2 Mb	27.0 Mb	23.2 Mb	27.2 Mb	21.2 Mb	25.2 Mb	21.3 Mb	26.6 Mb
Number of N50 Contig	13	11	12	11	12	11	12	11
Assembly size	593.0 Mb	593.0 Mb	595.3 Mb	595.3 Mb	578.4 Mb	578.4 Mb	576.7 Mb	576.7 Mb

Table S3 Summary statistics of chromosome lengths of male and female genome assemblies.

Chromosome	Length （bp)			
	Male diploid	Female diploid	Female_hap1 (Z)	Female_hap2 (W)
chr01	31,698,301	31,874,775	31,898,247	31,326,758
chr02	29,973,869	30,416,615	29,309,407	29,544,737
chr03	29,253,464	29,481,782	28,648,117	29,335,655
chr04	29,015,511	29,392,752	26,845,915	28,486,174
chr05	28,409,983	28,035,997	27,842,119	27,491,810
chr06	28,092,082	28,102,709	27,357,376	27,863,307
chr07	27,931,199	28,467,754	28,480,155	27,782,909
chr08	27,709,968	28,207,322	27,692,817	27,372,035
chr09	27,502,372	27,348,117	27,222,309	26,605,110
chr10	27,346,129	27,308,747	25,183,223	27,176,426
chr11	27,028,915	27,187,930	26,833,189	26,690,211
chr12	24,497,877	24,881,352	24,789,995	23,925,619
chr13	24,400,144	24,681,334	24,562,947	23,752,968
chr14	24,316,494	24,143,231	17,924,190	24,180,854
chr15	23,240,419	23,262,889	22,937,053	22,743,392
chr16	23,119,225	23,130,199	23,099,521	22,391,764
chr17	22,895,816	22,857,420	22,870,550	20,520,633
chr18	21,966,504	22,270,784	21,836,673	21,935,477
chr19	21,712,641	21,922,687	21,766,192	21,394,412
chr20	20,254,542	21,500,734	21,615,884	18,658,745
chr21	19,804,794	19,970,205	19,405,501	19,707,187
chr22	18,466,670	18,623,095	18,648,706	17,933,805
chr23	16,910,868	17,029,464	15,813,453	15,257,501
chr24	15,405,632	14,227,170	14,289,453	13,645,647
unchrs	2,111,558 (0.36 %)	976,364 (0.16 %)	1,515,315 (0.26%)	964,444(0.16 %)
Total	593,064,977	595,301,428	578,388,307	576,687,580

Table S4 Assessment of the genome assemblies for male and female samples and predicted gene models for male genome assembly by mapping to BUSCO database.

BUSCO assessment	Male (ZZ) %		Female (ZW) %		
	Diploid genome	Gene models	Diploid genome	Hap 1	Hap2
Total BUSCO groups searched (3640):					
Complete BUSCOs (C):	98.70	96.50	98.50	95.00	96.00
Complete and single-copy BUSCOs (S):	97.70	91.90	96.70	94.00	94.90
Complete and duplicated BUSCOs (D):	1.00	4.60	1.80	1.00	1.10
Fragmented BUSCOs (F):	0.30	1.60	0.30	0.30	0.40
Missing BUSCOs (M):	1.00	1.90	1.20	4.70	3.60

Table S5 Summary statistics of the sequence repeat content in the genome of ZZ genotype.

Classifications	No. elements	Length (bp)	Percentage
SINEs:	14,178	1,843,653	0.31%
ALUs	0	0	0%
MIRs	2,192	313,313	0.05%
LINEs:	26,478	10,578,433	1.78%
LINE1	1,504	910,020	0.15%
LINE2	10,876	3,317,641	0.56%
L3/CR1	0	0	0%
LTR elements:	7,347	1,808,106	0.30%
ERVL	0	0	0%
ERVL-MaLRs	0	0	0%
ERV_classI	1,884	471,022	0.08%
ERV_classII	0	0	0%
DNA elements:	129,108	27,561,613	4.65%
hAT-Charlie	6,159	1,154,768	0.19%
TcMar-Tigger	0	0	0%
Unclassified:	136,906	22,934,584	3.87%
Total interspersed repeats:		64,726,389	10.91%
Small RNA:	1863	995,044	0.17%
Satellites:	1236	875,561	0.15%
Simple repeats:	511,014	31,330,868	5.28%
Low complexity:	48,578	2,522,807	0.43%

Table S6 Positions and annotations of sex-specific variants.

Chr.	Position	Ref.	Alt.	Annotation
Chr1	30720112	G	T	Synonymous (*leo1*) [C/A]GA
Chr1	30724734	G	A	Intronic (*leo1*)
Chr1	30725492	C	T	Intronic (*leo1*)
Chr1	30725809	T	C	Intronic (*leo1*)
Chr1	30731652	A	G	Intronic (*tmod2*)
Chr1	30732516	C	T	Intronic (*tmod2*)
Chr1	30738353	G	T	Intronic (*tmod2*)
Chr1	30738363	G	A	Intronic (*tmod2*)
Chr1	30739025	G	A	Intronic (*tmod2*)
Chr1	30744282	C	T	Intergenic (*tmod2* /*lysmd2*)
Chr1	30745109	T	TTTAAAAA	Intergenic (*tmod2* /*lysmd2*)
Chr1	30745110	G	A	Intergenic (*tmod2* /*lysmd2*)
Chr1	30764840	G	A	Intergenic (*scg3* /*tnfaip8l3*)
Chr1	30766543	TACACTCACTCACACACAAT TAACTCCATGGAATATAA	T	Intergenic (*scg3* /*tnfaip8l3*)
Chr1	30772368	AACAGG	A	Intergenic (*scg3* /*tnfaip8l3*)
Chr1	30794837	A	T	Nonsynonymous (*cyp19a1a*) [A/T]CC
Chr1	30795197	T	C	Intergenic (*cyp19a1a* /*gldn*)
Chr1	30795240	C	G	Intergenic (*cyp19a1a* /*gldn*)
Chr1	30795282	CTG	C	Intergenic (*cyp19a1a* /*gldn*)
Chr1	30795749	T	TG	Intergenic (*cyp19a1a* /*gldn*)
Chr1	30795750	CTCACAGACTTTTCTCATGTT	C	Intergenic (*cyp19a1a* /*gldn*)
Chr1	30796090	C	T	Intergenic (*cyp19a1a* /*gldn*)
Chr1	30796198	C	CGCT	Intergenic (*cyp19a1a* /*gldn*)
Chr1	30796211	CCACGTGTTGGATAGAAA	C	Intergenic (*cyp19a1a* /*gldn*)
Chr1	30797870	GC	G	Intergenic (*cyp19a1a* /*gldn*)
Chr1	30799273	TGAAG	T	Intronic (*gldn*)
Chr1	30799634	C	T	Intronic (*gldn*)
Chr1	30801210	C	T	Synonymous (*gldn*) AC[C/T]
Chr1	30813539	G	A	Synonymous (*dmxl2*) AC[C/T]
Chr1	30813560	G	A	Synonymous (*dmxl2*) GG[C/T]

Table S7 Primer sequences used in this study.

Primer	Sequences	Annotation
Beta-actinF	TGCGTGACATCAAGGAGAAG	Beta-actin
Beta-actinR	ATGAATGACGGCTGGAAGAG	
InDel_1:30766543F	CATCTTGTGTACATTGGCCG	37 bp insertion 25kb downstream cyp19a1aZ
InDel_1:30766543R	GGACAAGTTCAGTGGGAGAGA	
InDel_1:30795750F	CTGAAAGTCAAACAGGGCCT	20 bp insertion ~ 800 bp upstream cyp19a1aZ & construction of luciferase vector
InDel_1:30795750R	ACGGACTGACTGGGAATTGT	
cyp19a1a.outer.q1F:	TGGTCGACATCTCCAACAGG	Detection cyp19a1a expression (nested PCR)
cyp19a1a.outer.q1R	GCGATCACCATCTCCAACAC	
cyp19a1a.inner.q2F	AAGGAGCTGCTGGTGAAGAT	Detection cyp19a1a expression (nested PCR)
cyp19a1a.inner.q2R	GCCGTGGTTCTGTGCAAATA	
W-cyp19a1aF:	TGGCAACCAGGACTCTCATC	Detection of W-linked cyp19a1a transcript
W-cyp19a1aR:	GACGCCCTGCTGATGATGA	
Z-cyp19a1aF:	CGCAGACACTCGTTGATGAA	Detection of Z-linked cyp19a1a transcript
Z-cyp19a1aR:	CAACAACAACGTCAGTCTGGA	
Insert.F2:	ACTCACCCGATGCAACAAAC	400 bp insertion upstream cyp19a1aZ
Insert.R2:	AGAAGAAGCAGCTCCTCTCC	
Var.Frag1_F1:	CTCTCTCTGACAAAGCTGCG	Construction of luciferase vector
Var.Frag1_R1:	AGAAGAAGCAGCTCCTCTCC	
